# Markedly different genome arrangements between serotype a strains and serotypes b or c strains of *Aggregatibacter actinomycetemcomitans*

**DOI:** 10.1186/1471-2164-11-489

**Published:** 2010-09-08

**Authors:** Weerayuth Kittichotirat, Roger Bumgarner, Casey Chen

**Affiliations:** 1 Department of Microbiology, University of Washington, Seattle, WA, USA; 2 Division of Periodontology, Diagnostic Sciences and Dental Hygiene, Herman Ostrow School of Dentistry of the University of Southern California, Los Angeles, CA, USA

## Abstract

**Background:**

Bacterial phenotype may be profoundly affected by the physical arrangement of their genes in the genome. The Gram-negative species *Aggregatibacter actinomycetemcomitans *is a major etiologic agent of human periodontitis. Individual clonal types of *A. actinomycetemcomitans *may exhibit variable virulence and different patterns of disease association. This study examined the genome arrangement of *A. actinomycetemcomitans *using the genome sequences of serotypes a-c strains. The genome alignment and rearrangement were analyzed by the MAUVE and the GRIMM algorithms. The distribution patterns of genes along the leading/lagging strands were investigated. The occurrence and the location of repeat sequences relative to the genome rearrangement breakpoints were also determined.

**Results:**

The genome arrangement of the serotype a strain D7S-1 is markedly different from the serotype b strain HK1651 or the serotype c strain D11S-1. Specific genome arrangements appear to be conserved among strains of the same serotypes. The reversal distance between D7S-1 and HK1651 by GRIMM analysis is also higher than the within-species comparisons of 7 randomly selected bacterial species. The locations of the orthologous genes are largely preserved between HK1651 and D11S-1 but not between D7S-1 and HK1651 (or D11S-1), irrespective of whether the genes are categorized as essential/nonessential or highly/nonhighly expressed. However, genome rearrangement did not disrupt the operons of the *A. actinomycetemcomitans *strains. A higher proportion of the genome in strain D7S-1 is occupied by repeat sequences than in strains HK1651 or D11S-1.

**Conclusion:**

The results suggest a significant evolutionary divergence between serotype a strains and serotypes b/c strains of *A. actinomycetemcomitans*. The distinct patterns of genome arrangement may suggest phenotypic differences between serotype a and serotypes b/c strains.

## Background

Bacterial genomes are relatively plastic and may display significant variation even among strains within the same species. The variation is often due to large scale genome deletion and/or gene acquisition by horizontal gene transfer of elements such as genomic islands [1]. Consequently, genome content can be divided into a core gene pool and a flexible gene pool [1-3]. The variation in genome content is thought to be a key factor in the evolution of bacterial pathogens. Moreover, the variation in genome arrangement (ie, the physical arrangement of genes) may also affect the virulence of the bacteria.

Genome rearrangement may occur via illegitimate recombination and homologous recombination among repeated elements and duplicated genes such as rDNA operons, and may also occur after horizontal gene transfer or phage infection. While genome rearrangements occurred frequently in laboratory cultures of *Escherichia coli*, very few were fixed since the divergence of *E. coli *and *Salmonella enterica *~100MYA [4,5]. Most of the rearrangements presumably have adverse effects on the bacteria due to the constraints placed by cellular processes such as replication, transcription and gene regulation [6,7]. Consequently, the genome rearrangements between closely related bacteria commonly involve large-scale inversions along the axis of the origin (Ori) and the terminus (Ter) of replication [8-10]. Such changes presumably have much less deleterious effects due to preservation of the gene locations relative to replication and other cellular processes.

Gram-negative facultative *Aggregatibacter actinomycetemcomitans *is a member of the *Pasteurellaceae *family [11]. It is a recognized pathogen in periodontitis and extra-oral infections. There are 6 distinct serotypes; each serotype may represent a distinct clonal lineage of *A. actinomycetemcomitans. *Depending on the disease status and race/ethnicity of the subjects dominant serotypes within the study populations may include serotypes a, b, c, and e [12,13]. Serotypes d and f are in general detected less frequently [12,13].

Certain clonal lineages of *A. actinomycetemcomitans*, such as the JP-2 clone, appear to exhibit a high degree of virulence [14-20]. However, other non-JP2 *A. actinomycetemcomitans *strains were also associated with aggressive periodontitis and are presumed to be highly virulent as well [13,21]. Interestingly, in the study of a subgingival microbial community by Socransky et al, *A. actinomycetemcomitans *serotype a strains were a component of the green complex, while *A. actinomycetemcomitans *serotype b strains were not in association with other bacterial species [22]. It seems plausible that *A. actinomycetemcomitans *strains are distinct in their phenotypes, pathogenic mechanisms, and functional roles in the subgingival microbial communities, which may result in different patterns of disease association.

To understand the molecular basis of the variations of virulence in *A. actinomycetemcomitans*, we sequenced and compared the genome content and structure of *A. actinomycetemcomitans *strains recovered from different clinical settings. We have obtained initial evidence for significant genome content variations among strains [23,24]. This study further examined the differences in the genome arrangement among *A. actinomycetemcomitans *strains of serotypes a-c. The results showed striking differences in the genome arrangements of serotype a strains compared to serotypes b or c strains. Such differences indicate divergent evolutionary pathways and possibly phenotypic differences between serotype a and serotype b/c strains of *A. actinomycetemcomitans.*

## Results

### Genome rearrangement between *A. actinomycetemcomitans *strains

**Figure 1:**
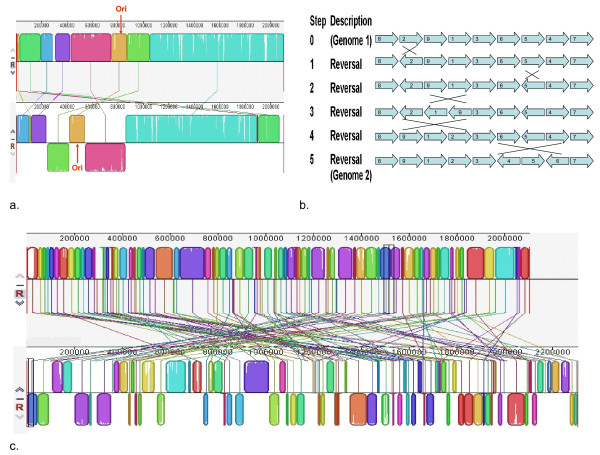
**Genome alignment by the progressive MAUVE between *A. actinomycetemcomitans *strains**. The program identifies stretches of nucleotide matches and selects locally collinear blocks (LCB) that meet a minimum weight criteria. Homologous LCBs between genomes are connected with a line and identified by the same color. Blocks that are inverted are placed under the center line of the genome. (a) Serotype b strain HK1651 (upper) and serotype c strain D11S-1 (lower). Nine LCB were identified. The predicted locations of Ori are indicated with red arrows. These two genomes are largely in synteny, which is also indicated by a relatively low reversal distance of 5 (see Table 1). The rearrangement between the genomes possibly involves an inversion along the Ori-Ter axis of the two LCBs flanking the Ori. (b) An optimal scenario of genome conversion between strain HK1651 and D11S-1 based on the GRIMM analysis. The LCBs are identified as numbered block arrows and also marked if they are involved in reversal in each step. The illustration merely gives an example and does not imply that the sequence of the inversions has to be the same as depicted. The result suggests that it is possible to convert one genome to the other by 5 steps of genome reversion. (c) Genome alignment of serotype b strain HK1651 (upper) and serotype a strain D7S-1 (lower). One hundred and two LCB were identified. These two genomes show little resemblance in their arrangements, which is also reflected by a relatively high reversal distance of 80 (see Table 1).

The results of genome comparison by MAUVE for *A. actinomycetemcomitans *are shown in Figure 1. The reversal distances obtained by GRIMM (for *A. actinomycetemcomitans *and other bacterial species) are summarized in Table 1. For comparison between D11S-1 and HK1651 there are 9 locally collinear blocks (LCBs) with a minimum weight of 8,386 identified by the progressive MAUVE (Figure 1a), with a reversal distance of 5 (Figure 1b). The reversal distance of 5 can be viewed as a hypothetical 5-step inversion process to convert the genome arrangement of one strain to the other strain. The rearrangements involved at least one large scale genomic inversion along the axis of Ori-Ter (see later section for more explanation). For the comparison between D7S-1 and HK1651 there are 102 LCBs with a minimum weight of 35 (Figure 1c) and a reversal distance of 80, which was not only greater than that between HK1651 and D11S-1 but also greater than those between strains in other bacterial species (Table 1).

**Table 1:** Genome rearrangement analysis and by progressive MAUVE and GRIMM of bacterial species.

Bacteria	**No. of LCB**^**a**^	**Reversal distance**^**b**^
*A. actinomycetemcomitans *HK1651 vs D7S-1	102	80
*A. actinomycetemcomitans *HK1651 vs D11S-1	9	5
*H. somnus *2336 vs 129PT	51	37
*H. influenzae *Rd KW20 vs 86-028NP	11	6
*H. influenzae *PittEE vs PittGG	23	17
*E. coli *536 vs ATCC8739	9	5
*E. coli *O157:H7 EDL933 vs O12:H6 E2348/69	9	7
*E. coli *O157:H7 EDL933 vs ATCC8739	6	3
*E. coli *CFT073 vs *E. coli *O157:H7 EDL933	21	16
*N. gonorrhoeae *NCCP11945 vs FA1090	14	10
*N. meningitides *FAM18 vs MC58	16	10
*N. meningitides *Z2491 vs MC58	14	8
*P. gingivalis *ATCC33277 vs W83	41	29
*P. aeruginosa *PA7 vs PAO1	12	7
*P. aeruginosa *LESB58 vs PAO1	8	5
*A. actinomycetemcomitans *HK1651 vs *A. aphrophilus *NJ8700	155	127
*A. actinomycetemcomitans *D7S-1 vs *A. aphrophilus *NJ8700	192	162

^a^LCB; locally colinear block identify by the progressive MAUVE

^b^Reversal distance is determined from the post-analysis of genome rearrangement by the progressive MAUVE using the GRIMM algorithms.

Seventy genome breakpoints of D7S-1 (in comparison to HK1651) were randomly selected for PCR analysis. All examined sites yielded PCR products of the expected sizes (see Additional File-1). Sixteen of the 70 PCR products were also sequenced and the results confirmed the sequences expected of the breakpoint regions (see Additional File-1).

### The conservation of genome structures within serotypes

A question may arise whether the genome arrangement of D7S-1 is unique and not found in other *A. actinomycetemcomitans *strains. To address this question we compared the genome arrangements of D7S-1, HK1651 and D11S-1 with those in the contigs of strains D17P-3, ANH9381, and D17P-2 (serotypes a, b and c, respectively). There were significantly fewer intra-contig breakpoints in the comparisons within each serotype than between serotypes a and b/c. We identified one intra-contig break point in 267 large contigs of D17P-3 in a pair-wise comparison to D7S-1. Similarly, we found 4 intra-contig breakpoints in 3 of the 102 large contigs of ANH9381 compared to HK1651, and 4 intra-contig breakpoints in 2 of the 62 large contigs of D17P-2 compared to D11S-1. In contrast, we identified 47 breakpoints in 40 contigs of D17P-3 compared to HK1651. The results are consistent with the conservation of the genome arrangement within serotypes, but not between serotypes a and b/c.

### Distribution patterns of genes and operons in the genomes of *A. actinomycetemcomitans*

It is possible that the relative gene locations in the genome may be preserved after large-scale genome rearrangements [6]. To address this question in *A. actinomycetemcomitans*, we first identified the Ori and the Ter in the genomes of D7S-1, HK1651 and D11S-1, and analyzed (i) the balance of the replichores, (ii) the gene density in the leading and the lagging strands, and (iii) the positions of the orthologous genes relative to the Ori. We further examined the preservation of the operons in strains of different genome arrangements.

The combined cumulative T-A and C-G skews of the 3^rd ^codons peaked at the nucleotide coordinate ~1,062 Kb and declined and changed sign at the nucleotide coordinate ~67 Kb for D7S-1 (see Additional File 2, top panel). Several peaks of similar heights were identified at coordinates 760 Kb, 825 Kb and 875 Kb for HK1651, and at 437 Kb, 515 Kb and 550 Kb for D11S-1 (see Additional File 2, middle and bottom panels). The lowest points of the combined skews changed sign at coordinates ~1,450 Kb for HK1651 and 1,246 Kb for D11S-1. Based on the results we assigned the Ori and the Ter respectively to nucleotide coordinates 1,062,100 and 67,100 in D7S-1, 825,100 and 1,449,500 in HK1651, and 515,300 and 1,246,100 in D11S-1. The predicted locations of Ori and Ter in HK1651 and D11S-1 were supported by the observation that a large-scale genomic inversion between HK1651 and D11S-1 occurred along the axis of Ori-Ter as commonly observed in other species [8-10].

The imbalance of the replichores was analyzed as described previously (the absolute value of [the length of the replichore-half length of the genome]/half length of the genome) [25]. We found that the imbalance of D7S-1 was 13.8%, which may be considered within the normal range of deviations among many bacterial species. In contrast, the imbalance of the replichores in HK1651 and D11S-1 were 40.1% and 30.6%, respectively.

The distributions of the predicted genes in the leading and the lagging strands are shown in Table 2. The gene density is higher in the leading strand than in the lagging strand in the 3 *A. actinomycetemcomitans *strains of this study. Higher numbers of essential genes were found in the leading strand than in the lagging strand for D7S-1 and HK1651, but D11S-1 showed no strand preference.

**Table 2:** Distribution of genes in leading and lagging strands in *A. actinomycetemcomitans *strains

Strain	Strand	Gene Density	No. of Essential Gene (%)	No. of Non-essential gene (%)
D7S-1	Leading	46.6%	153 (59.1)	1171 (52.2)
	Lagging	42.0%	106 (40.9)	1074 (47.8)
HK1651	Leading	46.2%	151 (56.8)	1124 (51.8)
	Lagging	43.9%	115 (43.2)	1047 (48.2)
D11S-1	Leading	45.1%	131 (50)	1007 (51.8)
	Lagging	43.5%	131 (50)	938 (48.2)

**Figure 2:**
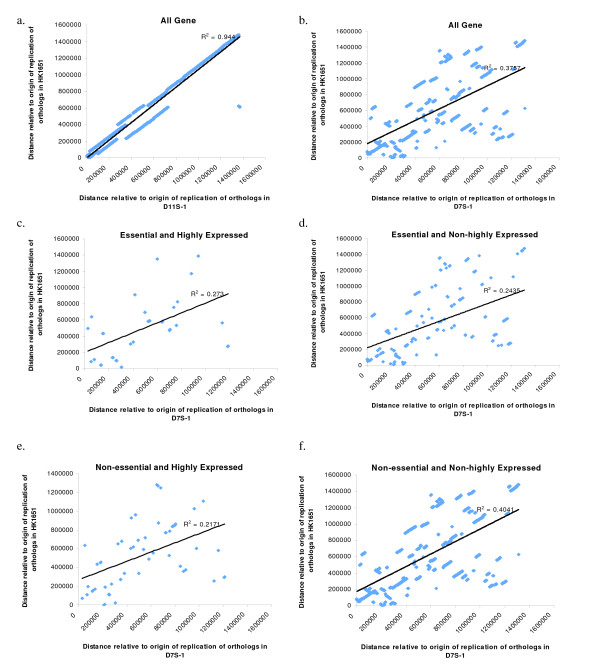
**Pair-wise comparisons of the distance to the origin of replication (Ori) for orthologous genes between *A. actinomycetemcomitans *strains**. The distances for genes on the leading strands were calculated directly from the position of Ori. The distances for genes on the lagging strands were calculated as (genome length-the distance to Ori). The linear trend line by Pearson's correlation is provided. The low r^2 ^values indicate a lack of correlation in the gene positions between D7S-1 and HK1651. (a) Comparison between HK1651 and D11S-1. There is an apparent correlation in the locations of orthologous genes in the strains. (b) Comparison between D7S-1 and HK1651. Although there is a hint of an overall linear relationship, the distribution pattern suggested that the locations of the orthologous genes were poorly correlated. (c-e) Comparisons between D7S-1 and HK1651 with genes of different categories. Again, little correlations were found in the locations of the orthologous genes between these strains.

The distances of the orthologous genes to the Ori are presented in Figure 2a-f. In Figure 2a the distances of each pair of the orthologous genes in HK1651 and D11S-1 were similar and can be explained by an offset of ~130 Kb between the genomes (with the exception of 8 genes where the differences in the distances to Ori were ~740 Kb). In contrast, the orthologous genes in HK1651 and D7S-1 (Figure 2b) resided in different locations relative to Ori and no specific distribution patterns were found. Similar results were found for subgroups of essential/nonessential and highly expressed/nonhighly expressed genes (Figure 2 c-f). Also, there was no discernable tendency for the highly expressed genes to be closer to the Ori than the non-essential genes.

The potential disruptions of operons in the *A. actinomycetemcomitans *strains were examined. The analysis of operon positions with respect to genomic rearrangement between D7S-1 and HK1651 showed that out of 564 operons in the D7S-1 genome predicted by Database of prOkaryotic OpeRons (DOOR) tool, 558 (98.9%) were found to be intact in HK1651 and were not affected by the genomic rearrangements between these strains (see Additional File 3 for the list of disrupted operons in D7S-1). Similarly, 505 of 515 (98%) predicted operons in HK1651 were found to be intact in D7S-1 (see Additional File 3 for the list of disrupted operons in HK1651). For the affected operons, the rearrangement breakpoints occurred between genes of the operons resulting in separations of genes rather than splitting the genes into two fragments. Similar results were obtained using FGENESB (http://linux1.softberry.com/berry.phtml?topic=fgenesb&group=programs&subgroup=gfindb) (data not shown).

### Features of genome rearrangement breakpoints

**Figure 3:**
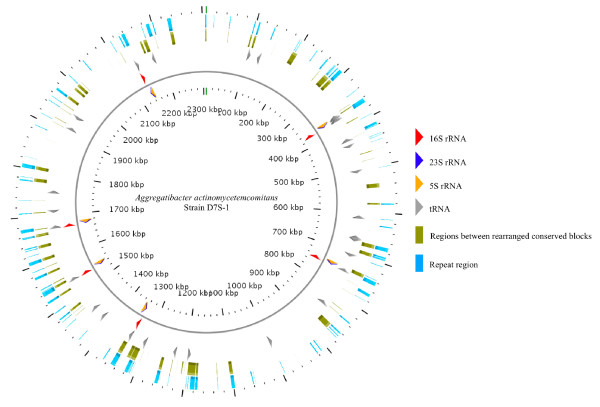
**Circular chromosome map of D7S-1 genome**. This figure shows that boundaries of the rearranged regions, as suggested by whole genome alignment of D7S-1 and HK1651 genome sequences, are significantly enriched with repeated sequences.

Genome rearrangements commonly occur via recombination between repeat elements or duplicated genes. We hypothesized that there might be specific features at the inter-LCBs regions and/or the ends of the LCBs that flanked the rearrangement breakpoints. To examine this hypothesis, 50-bp sub-sequences on both strands of each genome were extracted in sliding windows of 1 bp, and compared to the entire genome to identify a perfect match in other regions. These 50-base-pair-repeat regions are summarized in Table 3. D7S-1 genome contained a higher number of repeat regions than in HK1651, D11S-1 or other bacteria species analyzed. The percentage of the overlap between the cumulative inter-LCB regions and repeat regions are presented in Table 4. Higher percentages of the genome in D7S-1 were occupied by repeat regions than in the other two strains. Figure 3 illustrates the locations of the repeat elements and the inter-LCB breakpoint regions (relative to HK1651) of D7S-1. The regions between LCBs in D7S-1 were enriched with repeated sequences (See Additional File 4 for the locations of the repeat elements and the inter-LCB breakpoint regions of HK1651).

**Table 3:** Positions and percent of genome occupied by 50 base pair repeat in the genomes of *A. actinomycetemcomitans *and other bacterial species.

Genome	Total number of position with 50 bp exact matches	Genome length (bp)	Percent of 50 bp exact matches with respect to the genome length
D7S-1	144,825	2,308,328	6.27%
HK1651	44,531	2,105,503	2.11%
D11S-1	47,809	2,105,764	2.27%
*E. coli *K12	102,642	4,639,675	2.21%
*P. aeruginosa *PAO1	84,973	6,264,404	1.36%
*H. pylori *G28	41,316	1,652,982	2.50%

**Table 4:** Overlap between repeat and inter-LCB region

Strain	Total inter-LCB length (bp)	Total repeat length (bp)	Total overlap (bp)	Percent of repeat overlapping with inter-LCB	Percent of inter-LCB overlapping with repeat
D7S-1^a^	251,896	159,531	105,889	66.4%	42%
HK1651^b^	90,658	50,603	16,729	33.1%	18.5%
D11S-1^c^	112,883	53,208	20,973	39.4%	18.6%

^a ^Regions between rearranged conserved blocks (inter-LCB) for D7S-1 are obtained from D7S-1 vs HK1651 MAUVE alignment.

^b ^Regions between rearranged conserved blocks (inter-LCB) for HK1651 are obtained from D7S-1 vs HK1651 MAUVE alignment

^c ^Regions between rearranged conserved blocks (inter-LCB) for D11S-1 are obtained from D7S-1 vs D11S-1 MAUVE alignment

A summary comparison of the frequency and the feature of the repeat elements among strains are shown in Table 5 (see Additional File 5 for sequences of the repeat elements). D7S-1 has more repeat elements than HK1651 or D11S-1. Several of these repeat elements are shared among strains (allowing minor sequence variations). However, many of the repeat elements found in D7S-1 are unique to this strain.

**Table 5:** Frequency and features of the repeat elements identified in *A. actinomycetemcomitans*

Repeat ID	Estimated length (bp)	Features	Frequency in D7S-1 Genome	Frequency in HK1651 Genome	Frequency in D11S-1 Genome
REPEAT01	6505	rRNA genes operon	6	6	6
REPEAT02	1269	IS150 like element	8	7	5
REPEAT03	717	IS200 like element	0	4	5
REPEAT04	139	Unknown	1	5	5
REPEAT05	6326	FHA domain protein	2	0	0
REPEAT06	4315	WD-40 repeat, Hypothetical protein and FHA domain protein	2	0	0
REPEAT07	2156	Sel1 domain protein repeat-containing protein	4	0	0
REPEAT08	2008	Rhs element Vgr protein	6	0	0
REPEAT09	1523	Glycoside hydrolase, family 19 gene	5	0	0
REPEAT10	1254	FHA domain protein	2	0	0
REPEAT11	1227	Tra5 Protein	6	0	0
REPEAT12	1060	IS30 like element	3	0	0
REPEAT13	1225	Translation elongation factor Tu	2	2	2
REPEAT14	837	Putative FHA domain protein	4	0	0
REPEAT15	690	IS427 like element	4	0	0
REPEAT16	472	Sel1 domain protein repeat-containing protein	7	0	0
REPEAT17	387	Hypothetical protein	10	0	0
REPEAT18	219	Unknown	8	4	2
REPEAT19	162	Hypothetical protein	7	1	1
REPEAT20	100	Unknown	7	5	5
REPEAT21	135	Autotransporter adhesin Aae	4	3	2

## Discussion

A number of studies have suggested variable virulence among *A. actinomycetemcomitans *strains [12,15,16,19,20,26,27]. Most of these studies examined the clinical associations of specific genotypes of *A. actinomycetemcomitans *with periodontal health and disease in cross-sectional and prospective studies, but did not provide insight to the molecular basis of such variations. In the present study we provided evidence for variations in the physical arrangement of genes in the genomes of *A. actinomycetemcomitans*, which may affect the phenotypes or virulence of the strains. Serotypes a-c of *A. actinomycetemcomitans *were selected for this study because they are frequently identified and may represent up to 80% of the *A. actinomycetemcomitans *clinical isolates in human subgingival plaque [13].

The differences in the genome arrangement between strains were visualized with the use of MAUVE and then quantified by GRIMM to calculate the reversal distance. The use of reversal distance in phylogenetic analysis is based on the premise that genomic inversion is the primary type of rearrangement event in bacteria, which was supported by several studies [8,28]. Moreover, there is a general correlation between reversal distance and sequence-based phylogenetic analysis [28]. While low reversal distance may be relatively accurate, high reversal distance is likely to underestimate the true phylogenetic distance between genomes.

It is striking and unusual (in comparison to the variations seen in other bacterial species) that the serotype a strain D7S-1 displayed a markedly different genome arrangement relative to HK1651 or D11S-1. The potential sources of errors were examined first. We could rule out large scale sequencing or assembly errors of the contigs based on the results of the PCR analysis of the breakpoints in D7S-1. Moreover, minor sequencing errors would have little or no effect on the genome comparison by MAUVE which examines large homologous blocks between strains. We could also rule out assembly errors because the finished genomes were confirmed with optical mapping [23,24]. The results would not have been affected by the specific locations of the Ori or the Ter in the strains. Additional supporting evidence was from the analysis of unscaffolded large contigs of 3 *A. actinomycetemcomitans *strains. Few intra-contig breakpoints were found between strains of the same serotypes, in contrast to the high numbers of intra-contig breakpoints between strains of serotype a and serotypes b/c. Therefore we concluded that serotype a strains exhibited markedly different genome arrangements compared to those in serotypes b/c strains, and further suggested that the genome arrangements were conserved within serotypes.

The Ori and the Ter may be identified by various in silico methods based on analyses of DNA asymmetry, distribution of DNA boxes and *dnaA *gene location [29-32]. The methods based on DNA asymmetry appear to be the most universal and have been used to identify the Ori and Ter of *H. influenzae *(a member of the *Pasteurellacea*) [32,33]. In the absence of experimental determination the locations of the Ori and the Ter of *A. actinomycetemcomitans *strains identified in this study were in agreement with the available evidence as discussed below.

We noted that in the annotation of HK1651 the origin of replication was not identified but the starting codon of the *dnaA *gene was assigned the first nucleotide coordinate of the genome. While in some species the location of the *dnaA *gene coincides with the origin of replication [29], this is not the case in *H. influenzae *and presumably not in *A. actinomycetemcomitans *either. Eriksen et al [34] showed evidence of intragenomic recombination in JP2 clone of *A. actinomycetemcomitans *via homologous recombination of the 6 rRNA operons and 7 IS150-like repeat elements. It is interesting to note that, with the exception of one case (recombination between IS*150*-2 and IS*150*-4), all recombinations occurred along the axis of Ori-Ter predicted in this study. In this study we also found an example of a large-scale genomic inversion between HK1651 and D11S-1 along the axis of Ori-Ter.

Genome rearrangements may occur via homologous recombination of repeat elements in the bacterial genomes [35,36]. *A. actinomycetemcomitans *genomes contain diverse repeat elements that may mediate genome rearrangement. Within *A. actinomycetemcomitans *genomes some inter-LCB regions were occupied by composites of diverse repeat elements, which may reoccur in other inter-LCB regions but with minor variations (sequence variations, truncation, or absence) of the individual repeat elements. Some of the sequence diversity may be due to sequencing errors. For these reasons we chose to analyze the occurrence of repeat elements with a sliding 50-base window. We noted that *A. actinomycetemcomitans *D7S-1 has a greater number of repeat elements than HK1651 or D11S-1. The data alone, however, cannot be used to infer the ancestral genome structure of *A. actinomycetemcomitans.*

Some of the repeat elements are identified in all three *A. actinomycetemcomitans *strains. The IS150 like elements have been reported previously in the genome of HK1651 [34] and are found in both D7S-1 and D11S-1 in this study. The presence of variable copy numbers of a 135-bp repeat sequence in the autotransporter adhesion gene *Aae *in different *A. actinomycetemcomitans *strains has been reported previously [37]. There seems to be a distinction in the distribution pattern of the repeat elements in D7S-1 in comparison to that in HK1651/D11S-1. For example, 12 of the repeat elements in D7S-1 are unique and not found in HK1651 or D11S-1. Vice versa the REPEAT03 is identified in HK1651 and D11S-1 but not in D7S-1. Also, the copy numbers of some of the repeat elements (REPEAT-04, -19, -20) are identical in HK1651 and D11S-1 and different from the copy numbers of the elements found in D7S-1. Further examination of other *A. actinomycetemcomitans *strains is needed to determine whether such distribution pattern has any phylogenetic significance.

The results from this study appear to suggest that the genome arrangement of *A. actinomycetemcomitans *strains may be less constrained by cellular processes than in other bacterial species. This could be explained by several factors. The growth rate of *A. actinomycetemcomitans *is comparatively low (doubling time of ~3-4 hrs in optimum laboratory growth conditions). There might be little or no gene dosage effects and problem of collisions between replication fork and RNA polymerase in slow-growing bacteria, which allow the bacteria to tolerate large-scale genomic rearrangements. The effective population size of some clonal lineages of *A. actinomycetemcomitans *(e.g., serotypes b and c) may be small, which allow these clones to persist in the population. It is also possible that serotypes b and c, as represented by HK1651 and D11S-1, are more recently evolved and have not had sufficient time to allow the mutation pressures to exert their effects. This interpretation is supported by the imbalanced genomes of HK1651 and D11S-1, which could be a consequence of recent changes of their genome arrangements.

While there are significant differences in the genome arrangements in D7S-1 and HK1651 (or D11S-1) they essentially did not affect the operons. However, the locations of orthologous genes were significantly different between D7S-1 and HK1651 (or D11S-1). Presumably such differences will affect the phenotypes of the strains. We further noted the replichores were severely unbalanced for strains HK1651 and D11S-1 and less so for strain D7S-1. Evidence has suggested a strong selection for bacteria with a balanced genome [38]. On the contrary, no evidence of natural selection for balanced genomes was found in the analysis of eight *Yersinia *genomes [25]. We have detected no significant differences in growth rate and biofilm formation under laboratory growth conditions among these 3 *A. actinomycetemcomitans *strains (unpublished data). The significance of genome arrangement to the phenotypes of *A. actinomycetemcomitans *remains to be elucidated.

The differences in the genome arrangement or genome content alone may not be sufficient to determine whether some *A. actinomycetemcomitans *strains should be designated a subspecies or even a new species. There appear to be no universally accepted concept and definition of bacterial species. With the advancement of bacterial genomics various approaches for species definition have been proposed that combine the analyses of the 16S rRNA gene sequence identity, DNA-DNA hybridization, percentage of the shared genes in the genome, the average nucleotide identity (ANI) of the shared genes and ecological factors [39,40]. Pair-wise comparison of the 16S rRNA gene sequences in strains D7S-1, HK1651 and D11S-1 showed >97.6% nucleotide identity, which is within the accepted working definition of all three being from the same species. We are analyzing the genome contents of the sequenced serotypes a-f strains to further address this question.

In addition to the potential biological impact of the observed large-scale genomic rearrangement between serotype a strains and serotype b/c strains, there are also implications of the rearrangement on a practical research level. In the early stages of our assembly and finishing of the D7S-1 genome we had hoped to use the HK1651 genome as a guide to assist in the ordering of contigs. However, the level of genomic rearrangement between these two strains negated the utility of HK1651 as a reference genome for the structure of D7S-1. Also, the variation in genome structure means that PCR products predicted in one strain may cross a breakpoint in another strain and hence will not be amplified in the other strain. It is unclear how frequently similar problems will arise in the sequencing and analysis of other bacterial genomes, but it is worth noting that at least in *A. actinomycetemcomitans *massive variation in genome structure between strains can lead to confusion in some kinds of analyses.

## Conclusions

*A. actinomycetemcomitans *serotype a strains display markedly different physical arrangement of genes in comparison to serotype b or c strains. This likely indicates significant differences in the evolutionary history between serotype a strains and serotype b/c strains. The results have provided significant insight to the evolutionary divergence of *A. actinomycetemcomitans *of different serotypes. Also, the serotype-specific genome arrangement patterns have practical application for future genome sequencing of *A. actinomycetemcomitans*.

## Methods

### Bacterial strains

Serotype a strains D7S-1, D17P-3, and serotype c strains D11S-1 and D17P-2 were cultivated from subgingival plaque of patients with aggressive periodontitis [13,41]. Serotype b strain ANH9381 was recovered from a subgingival plaque sample of a periodontally non-diseased subject. Species identity and serotypes were examined by a 16S rRNA-based PCR analysis and a serotype analysis by a PCR-method as described previously [42].

### Genome sequences

The genome sequencing of D7S-1 (one contig; genome size 2,308,328 bp) and D11S-1 (circularized; genome size 2,105,764 bp) were completed as described previously [23,24]. The genome information of the sequenced strain HK1651 (genome size 2,105,503 bp) is accessible from University of Oklahoma (http://www.genome.ou.edu/act.html) and Oralgen (http://www.oralgen.lanl.gov/oralgen/bacteria/aact/). Contigs generated by 454 sequencing of strains D17P-3 (25× coverage), ANH9381 (16X) and D17P-2 (28X) were also included in the analyses. Additional bacterial genome sequences were downloaded from Genbank for analyses that included *Haemophilus somnus *strains 2336 and 129PT, *Haemophilus influenzae *strains RdKW20, 86-028NP, PittEE and PittGG, *Escherichia coli *strains 536, ATCC8739, O157:H7 EDL933, O12:H6 E2348/69, CFT073 and K12, *Neisseria gonorrhoeae *NCCP11945 and FA1090, *Neisseria meningitidis *FAM18, MC58, Z2491, *Porphyromonas gingivalis *ATCC33277 and W83, *Pseudomonas aeruginosa *PA7, PAO1, LESB58, *Aggregatibacter aphrophilus *NJ8700, and *Helicobacter pylori *G28.

### Annotation and comparison of *A. actinomycetemcomitans *genomes

A gene prediction and annotation pipeline was put together to process the genome sequence data obtained from the Roche/454 platform and strain HK1651. The gene identification and functional annotation mostly followed the protocol developed by The Institute for Genomic Research (J. Craig Venter Institute). Specifically, protein-coding genes were identified using Glimmer3 software [43] with our custom modification of the predicted results. Similarly, rRNA and tRNA coding genes were identified by using Exonerate [44] and tRNAscanSE [45] softwares, respectively. The predicted genes were annotated by first comparing them to the HK1651 annotation using the NCBI BLAST software [46]. Genes that are annotated as hypothetical as well as those that are not present in strain HK1651 were then blasted against Genbank non-redundant protein sequence database. The description of the best BLAST hit is then used as annotation for that gene. The gene orthologs among the 3 *A. actinomycetemcomitans *strains were identified based on all against all BLAST search. The genes that fulfill the following criteria are included as core genes with: (i) sequence similarity of at least 85% (ii) length difference of not more than 5%. Pseudogenes and genes with frameshift mutations were excluded from the analysis.

### Analysis of genomic rearrangement by MAUVE

The Progressive Mauve algorithm was used to create the whole genome alignment between different strains of *A. actinomycetemcomitans *[47]. GRIMM genome rearrangement algorithms were used to obtain the reversal distance between genomes [48].

### PCR analysis of genome breakpoints

The genome breakpoints in D7S-1 were analyzed by PCR. Briefly, 20-mer oligonucleotides were designed with the program Primer 3 [49]. A standard PCR protocol was employed under the following conditions: 5 min at 94°C for denaturation followed by 30 cycles of 94°C for 30 sec, an annealing step at 60°C for 1 min, an extension step at 72°C for 2 min and then a final extension of 10 min at 72°C [50]. PCR amplicons were analyzed by 1% agarose gel electrophoresis. For sequencing, the PCR products were purified by GIAquick PCR purification kit and GIAquick Gel Extraction kit (Qiagen, Valencia, CA) and submitted for sequencing at the USC School of Medicine Microchemical Core Facility.

### Assignment of gene categories in *A. actinomycetemcomitans*

All open reading frames (ORFs) identified in *A. actinomycetemcomitans *strains D7S-1, HK1651 and D11S-1 were categorized as essential or non-essential genes. The essential genes were identified based on the Profiling of *Escherichia coli *chromosome (PEC) database [51]. Specifically, *A. actinomycetemcomitans *ORFs that are homologous to the essential genes in PEC were considered as essential (blastp with E-value < = 1e-6). The remaining ORFs in *A. actinomycetemcomitans *were considered non-essential by default. ORFs were also classified into highly expressed and non-highly expressed genes based on the codon adaptation index (CAI) calculated using CAIJava tool [52]. ORFs within the top 5 percent highest CAI score are assigned as highly expressed and the remaining ORFs are considered as non-highly expressed.

### Analysis of gene density and gene positions

Combined G-C and T-A skews were first used to predict the locations of the Ori and the Ter with Oriloc [31]. Each genome was then divided into two replichores. The distribution of genes of different categories (essential/non-essential, highly expressed/non-highly expressed) and their densities in the leading and the lagging strands were compared between strains. The *Pearson's *correlation coefficient of the distance to the Ori for orthologous genes between strains was calculated.

### Analysis of preservation of operons among strains

The operons in *A. actinomycetemcomitans *were identified by using the Database of prOkaryotic OpeRons (DOOR) tool [53,54] and by FGENESB, a suite of bacterial operon and gene prediction programs [55]. In brief, the DOOR tool predicts bacterial gene operons using a classifier algorithm on features such as intergenic distance, neighborhood conservation, phylogenetic distance, information from short DNA motifs, similarity score between GO terms of gene pairs and length ratio between a pair of genes. The FGENESB tool predicts gene operon based on distances between open reading frames and frequencies of different genes neighboring each other in known bacterial genomes, as well as on promoter and terminator predictions. The positions of predicted operons in one strain were examined in the other strains to identify those that are affected by the genome rearrangement.

### Identification of regions with repeat sequences

A 50-base pair window (each window is a sliding of 1 base pair along the genome sequence) is compared to the entire genome to identify perfect matches (or a perfect match) on any other regions of the genome.

## Authors' contributions

WK participated in genome sequencing, gene annotations, identification of genome organization features, performed analysis of gene positions between strains, and identified and characterized repeated sequences of the genomes. RB led the efforts in genome sequencing, established and implemented the protocols for gene annotation and display, and led efforts in comparative genomic analysis between strains. CC conceived of the study and coordinated efforts of the project, performed genome arrangement analyses, and helped draft and finalized the manuscript. All authors contributed equally in data analysis and interpretation, and have read and approved the final manuscript.

## Supplementary Material

Additional file 1 **PDF PCR analysis of genome breakpoints of strain D7S-1**. The table provides the PCR primer sequences, the nucleotide coordinates of the PCR target sites in the genome of D7S-1, and the PCR results Click here for file

Additional file 2 **PDF Predicted Ori and Ter positions in *A. actinomycetemcomitans***. The figures show the T-A and C-G skew analysis of the genomes of strains D7S-1, HK1651 and D11S-1. Click here for file

Additional file 3 **PDF Predicted operons in *A. actinomycetemcomitans***. Two tables show the predicted operons in D7S-1 that are affected by the genomic rearrangement relative to HK1651 and vice versa. Click here for file

Additional file 4 **PDF Locations of repeat elements and inter-LCB regions in strain HK1651**. The figure shows the location of the repeat elements in the genome of HK1651 Click here for file

Additional file 5 **PDF Repeat elements in *A. actinomycetemcomitans***. The FASTA sequences of the repeat elements in *A. actinomycetemcomitans *strains D7S-1, HK1651 and D11S-1 Click here for file
