# Supplementary material for: Markedly different genome arrangements between serotype a strains and serotypes b or c strains of Aggregatibacter actinomycetemcomitans
Source: BMC Genomics. 2010 Sep 8;11:489. doi: 10.1186/1471-2164-11-489 (PMC2996985; doi:10.1186/1471-2164-11-489)
Supplement: Additional file 1 — PDF PCR analysis of genome breakpoints of strain D7S-1. The table provides the PCR primer sequences, the nucleotide coordinates of the PCR target sites in the genome of D7S-1, and the PCR results [file 1471-2164-11-489-S1.PDF]

Additional files

Additional File 1: PCR analysis of genome breakpoints of strain D7S-1

Primer Name	Left primer sequence 5'->3'	Right primer sequence 5'->3'	AMPLICON SIZE (b.p.)	Nucleotide Coordinate Start	Nucleotide Coordinate Stop	PCR Result	Sequencing result
16-1	CTGGATTGGACGGAATTTGT	GCCACGTACCAGCGTAATTT	1215	2011360	2012574	+	N/A
16-2	TGGAGCAGGAAAGGAAGAAA	TCACGCCCTCCGTTATTTAC	1248	2005643	2006890	+	N/A
23-1	GACAGCGACACCGTGAATAA	CCGTTTAAAACTGGCGTTGT	1176	2115998	2117173	+	N/A
23-2	CGCTTCCCGTCAATGTTATT	GCAAATTTGATGTGGTGCTG	986	2101906	2102891	+	N/A
23-3	CGCCACCATTTGTTCTTTTT	AATTTGGTTCCGTTCACAGC	851	2101583	2102433	+	N/A
86-1	ACGGGGATCTGTTGCACTAC	TGTTCATTCACGATCCGAAA	1231	823134	824364	+	N/A
86-2	AGTGGTAGCGAAATCCATCG	CAACAAGCCCTCCTAACGAA	763	784066	784828	+	N/A
246-1	TCTCGGCGACTAAGTTGGTT	TATCAAACGCGCCATGATTA	757	1299780	1300536	+	N/A
246-2	GTTTGAAAGTCACCGGGCTA	GACAACGTGATGGTGAATGC	364	1288341	1288704	+	N/A
255-1	TACCACCGGGACAAGAAGTC	GCGCGTAATTTTCACGATTT	801	2177740	2178540	+	N/A
255-2	GTTATCGCCTCCTCCACGTA	ACTGGAAGAAGGCACCAATG	895	2170697	2171591	+	N/A
255-3	CCTTGGGCCTTTTACCTTTT	CCAGAATAAGCCGGTAACCA	1035	2166561	2167595	+	N/A
255-4	AGATTCTTCCGGGTTGGTTT	TGCGAATTCCCTTTGAAGAT	705	2162846	2163550	+	N/A
281-1	TTCCTGTTTAACCGGCAATC	GAAGCTCCGTGCAGTTTTTC	504	1758139	1758642	+	N/A
285-1	CATCCACAGTATGAAGCGGA	TCGGGCGCGAAGACATCGGC	716	1016225	1016940	+	N/A
285-2	GTGCGGTCAAATATGAAACC	GGCGAAATGGAAGGTTTCGT	710	1015677	1016386	+	N/A
286-1	TGACGGGATAAACGGGTGAT	CCCGCACGAAATCATGCTCA	518	1638737	1639254	+	N/A
293-1	TGGAAACGTCCGGATTTTAG	ATTCACCAAAACCCAAGCTG	1595	1826211	1827805	+	N/A
293-2	CGGTAATGCACAACAAGTGG	AAAACCGCACAGGAATTACG	709	1869243	1869951	+	N/A
309-1	CCTATTTTCGGCAAAACCAA	AAACCGGCAAAGTTATCGTG	643	571591	572233	+	N/A
309-2	TCATCACCTCAATCCCCTTC	TTTCCCGCTATTCTGGTTTG	642	566469	567110	+	N/A
309-3	GTGGTGTTCGCGATAGGAAT	GGCTCCAGAATGGTGTGATT	714	545190	545903	+	N/A
309-4	ATTGACATCCTCAGGCGAAC	ATCCAAAGACCAGCCACATC	524	536308	536831	+	N/A
309-5	CCCTTGCATTATCGTTTGTG	AAAAATTTCCCCGTTTTCAC	538	515198	515735	+	N/A
309-6	ATTAGGCGACGGACATTTTG	TTTTGTAAGGTTCGGGTTCC	767	507710	508476	+	N/A
309-7	GCCGGATGAAGTGATTGTAG	TAGCCTTACTCCTCGCACTC	1020	482432	483451	+	N/A
309-8	TAACAACGCCACGGTTACAC	AAATCAATAACAGCGCCAAG	1877	465355	467231	+	N/A
309-9	AGCCAAAGGCAAAGCTATCG	AAGTGGCGGCACAATTAAAA	769	451505	452273	+	N/A
309-10	CCGTTGCCTTGAACATTATT	CTTTGCCGGCTACCAAATTA	1108	436788	437895	+	N/A
311-1	GATTCGGTCAGCATCAATCA	GCGGGGGAAAGTGTGAGGAA	795	201942	202736	+	N/A
311-2	TAGGCAACAAGCTCACATAT	TTGCGATTTTATCACCTAGG	714	191693	192406	+	N/A
311-3	ACTGCTCTTTGTTTTAGCCG	GTTAGCGATTCGCAAGCCAT	798	151574	152371	+	N/A
311-4	AAGTGGGTGACAAAGTGCTG	CCGGGAATTCCGCCTTGATT	715	144231	144945	+	N/A
311-5	AACTCATCGTTTACACCTCC	AATAATGGCGGCAGGGCTTG	671	140311	140981	+	N/A
314-1	ATTTTGTGGCGAACGGTAAG	GTGCTGCAACGCTATGAAAA	813	2196662	2197474	+	N/A
320-1	TGCGGTATCATTCAGCACTT	ACATTTGATTGAGGGCAAGC	754	388141	388894	+	N/A
320-2	GGAAATTCAACGCATCACAA	AAGCGGGTTGGTGTATCAAG	667	389540	390206	+	N/A
321-1	GCGATACGCAGGACAAAAAT	CAACGGCAATGTGATTCAAG	1343	1467774	1469116	+	confirmed
323-1	TCGTTATTCGGGTCGAAATC	GCTCCAGTCGCTTAAAATCC	861	1165240	1166100	+	confirmed
323-2	GGCGAAACCAATGAGGAAAT	CCGACACGCCGTACACCACA	1360	1130607	1131966	+	confirmed
326-1	ACGGTAAAAATTCGCCAGTG	GCATTAAGCGCAACATTGAA	667	1723376	1724042	+	confirmed
342-1	TTAATGGAAACGGAAGGATG	CGAGGCTTTGCAATTTGAAC	800	68627	69426	+	N/A
345-1	CTTGTGCGAATTCTGTTAGA	TCCCACTTCGGTACAGCTTC	880	1194473	1195352	+	confirmed
345-2	TAGCGGAGATGATTCGGTTT	TCACCACCGTACAAAGCATC	888	1201540	1202427	+	confirmed
345-3	TCTCAGCGTCATCCAACAAG	CGGCACAATACCAAATGTCA	849	1206451	1207299	+	confirmed

345-4	CTGGGATCTGAACTGCGTTT	CACCTCCCGTTCCAATTTAG	1280	1218752	1220031	+	confirmed
345-5	TCAAGCCATGTGGGTGAATA	ATTGATTCATCGCCGATTTC	1385	1230404	1231788	+	confirmed
346-1	GAGGTGAGGCAATGAATACA	TACGCAGGGGATTTAACTTG	870	1946680	1947549	+	N/A
346-2	GCCCATGTTGGAACCTGCCA	TTGCAATATCTCACTGCAGC	1576	1941750	1943325	+	N/A
346-3	CTGGACCTAAAATATTCGTA	GCAAGTGGAAGTACCGCCTC	801	1919306	1920106	+	N/A
346-4	TGACTATTGATGTTGTGGGT	GATATGGAAGCGTTGTGCAG	864	1918486	1919349	+	N/A
346-5	TTATTTTCCTTGTATTATCC	TGCCCGTTTTGCAGAAATTG	1038	1908524	1909561	+	N/A
360-1	TCGATTTCTTTGCTATTTAA	ATAGCACAACAAAGTGCGGT	782	1529621	1530402	+	N/A
365-1	GCAGCCCCGTTATTATTTCT	CCCGTCCAGCTTGTATTGGT	2356	724023	726378	+	N/A
365-2	GATGGCAAATGGGTGATTAC	TTGCTTGTCGCAAGAACAAA	789	717525	718313	+	N/A
365-3	AATGCGCGTAGATCACAACG	ATTAAGTCGTTTCCGCATTC	576	712694	713269	+	N/A
380-1	ATTTTCGTACAAGGCGTGCT	GCAATCCGCTAAAAATTGGA	615	2246036	2246650	+	N/A
380-2	CATTTTCACACTGCGTCCAT	CTTCCGCACCTTTATTACCG	790	2247282	2248071	+	N/A
380-3	CGCCAGAAGAAGGAAAGTTG	CATCAGCCAAATTCAGGGAT	678	2253209	2253886	+	N/A
380-4	CTTTTGCCGGTGTAGAAAGC	CTCCTAGCCATAGCCGTTTG	1033	2273059	2274091	+	confirmed
380-5	GCATTTGACGGGATTTCTTG	CCACCAAAATTGCCTTAGGA	760	2292849	2293608	+	confirmed
380-6	AAACGCCAAGTGGATATTCG	TACTGCCCAACAAACCTTCC	798	2300547	2301344	+	N/A
380-7	TTTGCGCCAGTAATTCTTCC	GATTATCATTTGGCGGAACG	775	2303815	2304589	+	confirmed
393-1	CCGTTGGCGTGTTCTAATTT	CCCACCGATTTATGATCACC	681	99983	100663	+	N/A
393-2	CCGTTGGCGTGTTCTAATTT	CCCACCGATTTATGATCACC	681	99983	100663	+	N/A
393-3	ATTTGGCTGTATGGCGGTAG	AGAAGAAAGTGCGGTGGAAA	768	97955	98722	+	N/A
394-1	AGCCCCTGAGTTATCAGCAA	CGACCTTCGCTTACCAAGTC	853	762496	763348	+	confirmed
394-2	GGGCAAGGGCTAACATAACA	TTTGTGGAAATTGGTGACGA	590	766031	766620	+	confirmed
395-1	TTCCCGTATGCTCGAAGTCT	GGGTGGACATAAAGATTTCC	539	1097030	1097568	+	confirmed
395-2	GGCATTGTGACCGTTTCTTT	AGAAGCGTTAGCCACGAAAA	601	1087273	1087873	+	confirmed
