# Supplementary material for: Markedly different genome arrangements between serotype a strains and serotypes b or c strains of Aggregatibacter actinomycetemcomitans
Source: BMC Genomics. 2010 Sep 8;11:489. doi: 10.1186/1471-2164-11-489 (PMC2996985; doi:10.1186/1471-2164-11-489)
Supplement: Additional file 2 — PDF Predicted Ori and Ter positions in A. actinomycetemcomitans. The figures show the T-A and C-G skew analysis of the genomes of strains D7S-1, HK1651 and D11S-1. [file 1471-2164-11-489-S2.PDF]

Additional files

Additional File 2: Predicted Ori and Ter positions in *A. actinomycetemcomitans*

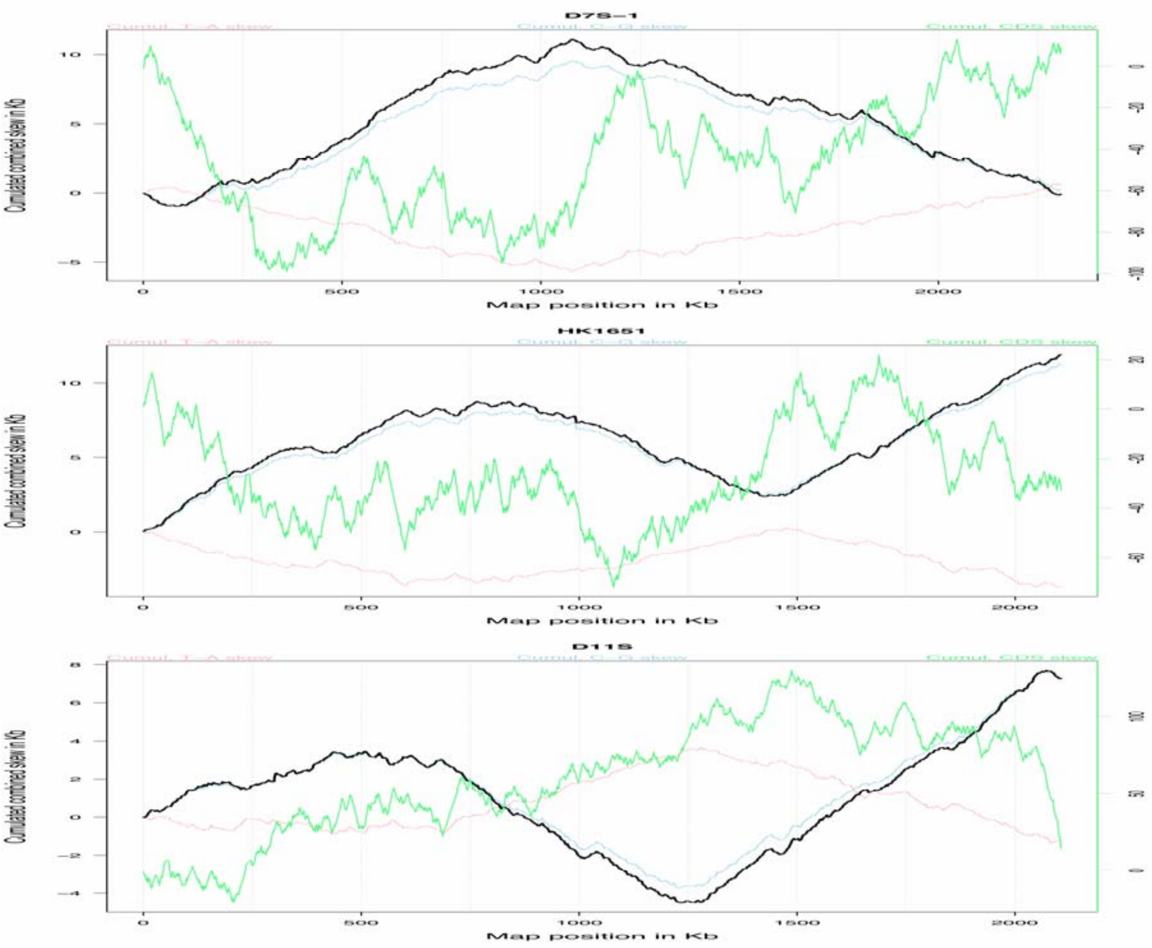

T-A, C-G and CDS skew analysis of the genomes of strains D7S-1, HK1651 and D11S-1. The black lines are combined signals from T-A and C-G skews and equivalent to analysis of “keto excess”. The peaks and the lowest points of keto excess coincide with the replication origin and termination in many bacterial species.
