# Supplementary material for: Markedly different genome arrangements between serotype a strains and serotypes b or c strains of Aggregatibacter actinomycetemcomitans
Source: BMC Genomics. 2010 Sep 8;11:489. doi: 10.1186/1471-2164-11-489 (PMC2996985; doi:10.1186/1471-2164-11-489)
Supplement: Additional file 3 — PDF Predicted operons in A. actinomycetemcomitans. Two tables show the predicted operons in D7S-1 that are affected by the genomic rearrangement relative to HK1651 and vice versa. [file 1471-2164-11-489-S3.PDF]

Additional files

Additional file 3 – Predicted operons in *A. actinomycetemcomitans*

Table A1. Predicted D7S-1 operons (by DOOR tool) that are affected by the genomic rearrangement relative to HK1651 genome.

-D7S-1 Predicted operon ID: OP529 (Coordinates 1,011,337-1,016,641)

Order in D7S-1

ORF01049	1011337	1011663	D7S_1087	DNA repair protein RecO
ORF01050	1011729	1012025	D7S_1088	DNA repair protein RecO
ORF01051	1012027	1013343	D7S_1089	RNA methyltransferase
ORF01052	1013353	1015584	D7S_1090	GTP pyrophosphokinase
ORF01053	1015610	1015966	D7S_1091	DgkA protein
ORF01054	1015982	1016641	D7S_1092	HAD hydrolase, family IA

Order in HK1651

AA01070	741060	741743	AA_0866	DNA repair protein RecO
AA01071	741847	743061	AA_0867	RNA methyltransferase
AA01072	743074	745302	AA_0868	GTP pyrophosphokinase
AA01074	745330	745683	AA_0869	DgkA protein
AA01270	863457	863957	AA_1039	HAD hydrolase, family IA

-**D7S-1 Predicted operon ID: OP837 (Coordinates 1,637,357-1,640,367)**

Order in D7S-1

ORF01735	1638292	1637357	D7S_1788	putative protein insertion permease FtsX
ORF01736	1638951	1638301	D7S_1789	cell division ATP-binding protein FtsE
ORF01737	1640162	1638990	D7S_1790	cell division protein FtsY
ORF01738	1640367	1640191	D7S_1791	cell division protein FtsY

Order in HK1651

AA01423	956808	957740	AA_1156	putative protein insertion permease FtsX
AA01422	956149	956796	AA_1155	cell division ATP-binding protein FtsE
AA00474	331784	330315	AA_0382	cell division protein FtsY

-D7S-1 Predicted operon ID: OP884 (Coordinates 1,723,043-1,725,202)

Order in D7S-1

ORF01832	1723627	1723043	D7S_1889	peptidyl-tRNA hydrolase
ORF01833	1724247	1723756	D7S_1890	nucleotide-binding protein
ORF01834	1725202	1724258	D7S_1891	phosphoserine phosphatase

Order in HK1651

AA02418	1661871	1661230	AA_1942	peptidyl-tRNA hydrolase
AA02937	2057007	2056519	AA_2378	nucleotide-binding protein
AA02939	2057962	2057021	AA_2379	phosphoserine phosphatase

-D7S-1 Predicted operon ID: OP895 (Coordinates 1,756,070-1,760,461)

Order in D7S-1

ORF01865	1756070	1758487	D7S_1923	ATP-dependent protease La
ORF01866	1758614	1760461	D7S_1924	SurA protein

Order in HK1651

AA02395	1644753	1647167	AA_1925	ATP-dependent protease La
AA00114	84031	82187	AA_0086	SurA protein

-**D7S-1 Predicted operon ID: OP1048 (Coordinates 2,085,468-2,089,149)**

Order in D7S-1

ORF02195	2085468	2086487	D7S_2257	lytic transglycosylase
ORF02196	2086505	2087308	D7S_2258	exonuclease III
ORF02197	2087308	2088096	D7S_2259	hypothetical protein
ORF02198	2088121	2089149	D7S_2260	dTDP-glucose 4,6-dehydratase

Order in HK1651

AA02648	1845697	1844570	AA_2141	lytic transglycosylase
AA02628	1830065	1829265	AA_2122	exonuclease III
AA02626	1829262	1828471	AA_2121	hypothetical protein
AA02623	1828039	1827440	AA_2120	dTDP-glucose 4,6-dehydratase

-D7S-1 Predicted operon ID: OP1158 (Coordinates 2,291,001-2,294,065)

Order in D7S-1

ORF02419	2291381	2291001	D7S_2485	Fe-S metabolism associated domain subfamily
ORF02420	2292577	2291378	D7S_2486	aminotransferase, class-V
ORF02421	2293083	2292583	D7S_2487	protein ParB
ORF02422	2294065	2293100	D7S_2488	threonyl-tRNA synthetase

Order in HK1651

AA02581	1797106	1797483	AA_2087	Fe-S metabolism associated domain subfamily
AA02579	1795913	1797106	AA_2086	aminotransferase, class-V
AA02577	1795407	1795904	AA_2085	protein ParB
AA02160	1477934	1478989	AA_1737	threonyl-tRNA synthetase

Table A2. Predicted HK1651 operons (by DOOR tool) that are affected by the genomic rearrangement relative to D7S-1 genome

-HK1651 Predicted operon ID: OP24 (Coordinates 66,658-71,233)

Order in HK1651

AA00089	66658	67809	AA_0070	methionine adenosyltransferase
AA00091	67881	68378	AA_0071	SprT protein
AA00092	68538	69776	AA_0072	serine transporter
AA00093	69872	71233	AA_0073	L-serine ammonia-lyase

Order in D7S-1

ORF01912	1799554	1800708	D7S_1970	methionine adenosyltransferase
ORF01883	1774690	1774190	D7S_1941	SprT protein
ORF01882	1774033	1772792	D7S_1940	serine transporter
ORF01881	1772699	1771335	D7S_1939	L-serine ammonia-lyase

-**HK1651 Predicted operon ID: OP29 (Coordinates 80,049-86,437)**

Order in HK1651

AA00112	81299	80049	AA_0084	UraA protein
AA00113	82049	81426	AA_0085	uracil phosphoribosyltransferase
AA00114	84031	82187	AA_0086	SurA protein
AA00115	86437	84167	AA_0087	glutamate--cysteine ligase/gamma-glutamylcysteine synthetase

Order in D7S-1

ORF01868	1761346	1762599	D7S_1926	UraA protein
ORF01867	1760596	1761222	D7S_1925	uracil phosphoribosyltransferase
ORF01866	1758614	1760461	D7S_1924	SurA protein
ORF01674	1579963	1577690	D7S_1727	glutamate--cysteine ligase/gamma-glutamylcysteine synthetase

-HK1651 Predicted operon ID: OP51 (Coordinates 112,976-117,979)

Order in HK1651

AA00160	112976	113494	AA_0123	putative fimbrial subunit PilA
AA00161	113524	114930	AA_0124	type IV pilus assembly protein
AA00162	114926	116146	AA_0125	putative type IV pilin secretion protein
AA00163	116146	116832	AA_0126	prepilin peptidase
AA00164	116885	117505	AA_0127	dephospho-CoA kinase
AA00165	117498	117707	AA_0128	dephospho-CoA kinase
AA00166	117695	117979	AA_0129	acetyltransferase

Order in D7S-1

ORF01711	1613349	1612828	D7S_1764	putative fimbrial subunit PilA
ORF01710	1612801	1611392	D7S_1763	type IV pilus assembly protein
ORF01709	1611399	1610176	D7S_1762	putative type IV pilin secretion protein
ORF01708	1610176	1609490	D7S_1761	prepilin peptidase
ORF01667	1573412	1572789	D7S_1720	dephospho-CoA kinase
ORF01666	1572799	1572587	D7S_1719	dephospho-CoA kinase
ORF01665	1572587	1572315	D7S_1718	acetyltransferase

-HK1651 Predicted operon ID: OP61 (Coordinates 135,685-138,804)

Order in HK1651

AA00194	137049	135685	AA_0154	oxygen-independent coproporphyrinogen III oxidase
AA00195	137502	137071	AA_0155	hypothetical protein
AA00196	138075	137518	AA_0156	hypothetical protein
AA00198	138703	138179	AA_0157	tRNA-specific adenosine deaminase
AA00199	138804	138715	AA_0158	hypothetical protein

Order in D7S-1

ORF01640	1553233	1554600	D7S_1693	oxygen-independent coproporphyrinogen III oxidase
ORF01639	1552780	1553214	D7S_1692	hypothetical protein
ORF01638	1552207	1552767	D7S_1691	hypothetical protein
ORF00706	695854	695327	D7S_0732	tRNA-specific adenosine deaminase

-HK1651 Predicted operon ID: OP104 (Coordinates 228,937-230,713)

Order in HK1651

AA00340	228937	229485	AA_0268	flavodoxin fold family protein
AA00341	229528	229863	AA_0269	YCII-related protein
AA00342	229835	229999	AA_0270	hypothetical protein
AA00343	230048	230713	AA_0271	Antibiotic biosynthesis monooxygenase

Order in D7S-1

ORF01568	1486194	1485868	D7S_1618	flavodoxin fold family protein
ORF01567	1485673	1485449	D7S_1617	YCII-related protein
ORF01699	1603623	1602919	D7S_1752	Antibiotic biosynthesis monooxygenase

-HK1651 Predicted operon ID: OP547 (Coordinates 1,134,496-1,149,202)

Order in HK1651

AA01679	1134496	1135296	AA_1356	aspartate-semialdehyde dehydrogenase
AA01681	1135299	1135730	AA_1357	molybdate ABC transporter, periplasmic molybdate-binding protein
AA01682	1135708	1136502	AA_1358	molybdate ABC transporter, permease
AA01684	1136442	1137119	AA_1359	sulfate/thiosulfate import ATP-binding protein CysA
AA01685	1137119	1137976	AA_1360	ModD protein
AA01686	1138053	1138196	AA_1361	hypothetical protein
AA01687	1138183	1139721	AA_1362	U62 family peptidase 2 TldD
AA01688	1139775	1145543	AA_1363	alpha-2-macroglobulin domain protein
AA01689	1145640	1146725	AA_1364	phosphoenolpyruvate carboxylase
AA01690	1146848	1149202	AA_1365	penicillin-binding protein 1C

Order in D7S-1

ORF02226	2115512	2116483	D7S_2288	aspartate-semialdehyde dehydrogenase
ORF02072	1953608	1954342	D7S_2134	molybdate ABC transporter, periplasmic molybdate-binding protein
ORF02073	1954311	1955114	D7S_2135	molybdate ABC transporter, permease

ORF02074	1955117	1955731	D7S_2136	sulfate/thiosulfate import ATP-binding protein CysA
ORF02075	1955728	1956588	D7S_2137	ModD protein
ORF02076	1956661	1956873	D7S_2138	hypothetical protein
ORF02077	1956885	1958333	D7S_2139	U62 family peptidase 2 TldD
ORF02078	1958399	1964170	D7S_2140	alpha-2-macroglobulin domain protein
ORF02079	1964304	1965353	D7S_2141	phosphoenolpyruvate carboxylase
ORF02080	1965473	1967830	D7S_2142	penicillin-binding protein 1C

-HK1651 Predicted operon ID: OP688 (Coordinates 1,416,241-1,429,016)

<u>Order in HK1651</u>

AA02082	1416241	1417908	AA_1673	ribonuclease G
AA02084	1417983	1418414	AA_1674	hypothetical protein
AA02086	1418517	1419074	AA_1675	4-alpha-glucanotransferase
AA02087	1419074	1421266	AA_1676	1,4-alpha-glucan branching enzyme
AA02088	1421422	1422153	AA_1677	glycogen debranching enzyme GlgX
AA02089	1422190	1423272	AA_1678	glycogen debranching enzyme GlgX
AA02090	1423302	1424609	AA_1679	glucose-1-phosphate adenylyltransferase
AA02091	1424581	1424727	AA_1680	hypothetical protein
AA02092	1424814	1426250	AA_1681	starch synthase
AA02094	1426376	1428838	AA_1682	glycogen synthase
AA02096	1428894	1429016	AA_1683	hypothetical protein
AA02097	1429565	1428963	AA_1684	cobalt transport protein CbiM

<u>Order in D7S-1</u>

ORF02351	2227993	2229684	D7S_2417	ribonuclease G
ORF02355	2230500	2230856	D7S_2421	hypothetical protein
ORF00086	95429	94860	D7S_0086	4-alpha-glucanotransferase
ORF00085	94872	92677	D7S_0085	1,4-alpha-glucan branching enzyme
ORF00084	92680	90671	D7S_0084	glycogen debranching enzyme GlgX
ORF00083	90644	89334	D7S_0083	glucose-1-phosphate adenylyltransferase
ORF00082	89132	87693	D7S_0082	starch synthase
ORF00081	87570	85105	D7S_0081	glycogen synthase
ORF00080	84940	85080	D7S_0080	hypothetical protein
ORF00079	84381	84986	D7S_0079	cobalt transport protein CbiM

-HK1651 Predicted operon ID: OP703 (Coordinates 1,461,301-1,469,537)

<u>Order in HK1651</u>

AA02139	1461301	1462278	AA_1717	AcrA protein
AA02140	1462284	1465025	AA_1718	CcmA protein
AA02141	1465030	1466154	AA_1719	ABC-2 type transporter
AA02142	1466177	1467592	AA_1720	TolC protein
AA02143	1467573	1467701	AA_1721	hypothetical protein
AA02144	1467723	1468739	AA_1722	HmuU protein
AA02145	1468742	1469236	AA_1723	ABC-type cobalamin/Fe3+-siderophore transport system, ATPase component
AA02146	1468968	1469537	AA_1724	ABC-type cobalamin/Fe3+-siderophore transport system, ATPase component

<u>Order in D7S-1</u>

ORF00056	54429	53449	D7S_0056	AcrA protein
ORF00055	53446	50702	D7S_0055	CcmA protein
ORF00054	50700	49573	D7S_0054	ABC-2 type transporter
ORF00053	49553	48135	D7S_0053	TolC protein
ORF00052	48125	48009	D7S_0052	hypothetical protein
ORF02434	2306001	2304937	D7S_2500	HmuU protein
ORF02433	2304937	2304143	D7S_2499	ABC-type cobalamin/Fe3+-siderophore transport system, ATPase component

-**HK1651 Predicted operon ID: OP830 (Coordinates 1,769,996-1,774,281)**

<u>Order in HK1651</u>

AA02541	1772704	1769996	AA_2054	putative type III restriction enzyme
AA02543	1773494	1772841	AA_2055	hypothetical protein
AA02544	1774005	1773457	AA_2056	putative DEAD/DEAH box helicase
AA02545	1774281	1774105	AA_2057	CRISPR-associated helicase Cas3 domain protein

<u>Order in D7S-1</u>

ORF02115	1997664	2000381	D7S_2177	putative type III restriction enzyme
ORF02109	1990330	1993206	D7S_2171	putative DEAD/DEAH box helicase
ORF02108	1988490	1990193	D7S_2170	crispr-associated helicase Cas3 domain protein

Note that AA_2055 does not exist in D7S-1

-HK1651 Predicted operon ID: OP861 (Coordinates 1,832,956-1,849,307)

<u>Order in HK1651</u>

AA02634	1833822	1832956	AA_2127	glycosyltransferase
AA02635	1834195	1833818	AA_2128	hypothetical protein
AA02636	1834899	1834198	AA_2129	putative glycosyltransferase
AA02636.1	1835294	1834911	AA_2130	dTDP-glucose-4-keto-6-deoxy-D-glucose reductase
AA02637	1836619	1835282	AA_2131	membrane protein-like protein
AA02638	1837298	1836609	AA_2132	NAD-dependent epimerase/dehydratase
AA02639	1838526	1837273	AA_2133	glycosyl transferase
AA02640	1839497	1838529	AA_2134	rhamnosyltrasferase
AA02642	1840120	1839497	AA_2135	ABC transport protein
AA02643	1841025	1840237	AA_2136	ABC transporter integral membrane subunit
AA02644	1841598	1841062	AA_2137	dTDP-4-keto-6-deoxy-D-glucose-3,5-epimerase
AA02645	1842479	1841604	AA_2138	dTDP-4-rhamnose reductase
AA02646	1843379	1842483	AA_2139	glucose-1-phosphate-thymidylyltransferase
AA02647	1844497	1843433	AA_2140	dTDP-D-glucose-4,6-dehydratase
AA02648	1845697	1844570	AA_2141	lytic transglycosylase
AA02649	1846484	1845702	AA_2142	amylovoran biosynthesis glycosyltransferase AmsE
AA02651	1847458	1846580	AA_2143	glycosyltransferase
AA02652	1848651	1847461	AA_2144	hypothetical protein
AA02654	1849307	1848663	AA_2145	Lsg locus putative protein 4

Order in D7S-1

ORF01284	1209076	1209828	D7S_1324	putative ABC-transporter
ORF01283	1208921	1209079	D7S_1323	putative ABC-transporter
ORF01282	1208289	1208915	D7S_1322	putative ABC-transporter
ORF02195	2085468	2086487	D7S_2257	lytic transglycosylase
ORF02194	2085478	2085332	D7S_2256	hypothetical protein
ORF02193	2084558	2085343	D7S_2255	amylovoran biosynthesis glycosyltransferase AmsE
ORF02192	2083584	2084465	D7S_2254	WcaA protein
ORF02191	2082394	2083584	D7S_2253	hypothetical protein
ORF02190	2081609	2082385	D7S_2252	Lsg locus putative protein 4

Note that AA_2127, AA_2128, AA_2129, AA_2130, AA_2131, AA_2132, AA_2133, 2134, AA_2137, AA_2138, AA_2139, AA_2140 do not exist in D7S-1
