# Supplementary material for: Markedly different genome arrangements between serotype a strains and serotypes b or c strains of Aggregatibacter actinomycetemcomitans
Source: BMC Genomics. 2010 Sep 8;11:489. doi: 10.1186/1471-2164-11-489 (PMC2996985; doi:10.1186/1471-2164-11-489)
Supplement: Additional file 4 — PDF Locations of repeat elements and inter-LCB regions in strain HK1651. The figure shows the location of the repeat elements in the genome of HK1651 [file 1471-2164-11-489-S4.PDF]

Additional files

Additional File 4: Locations of repeat elements and inter-LCB regions in strain HK1651

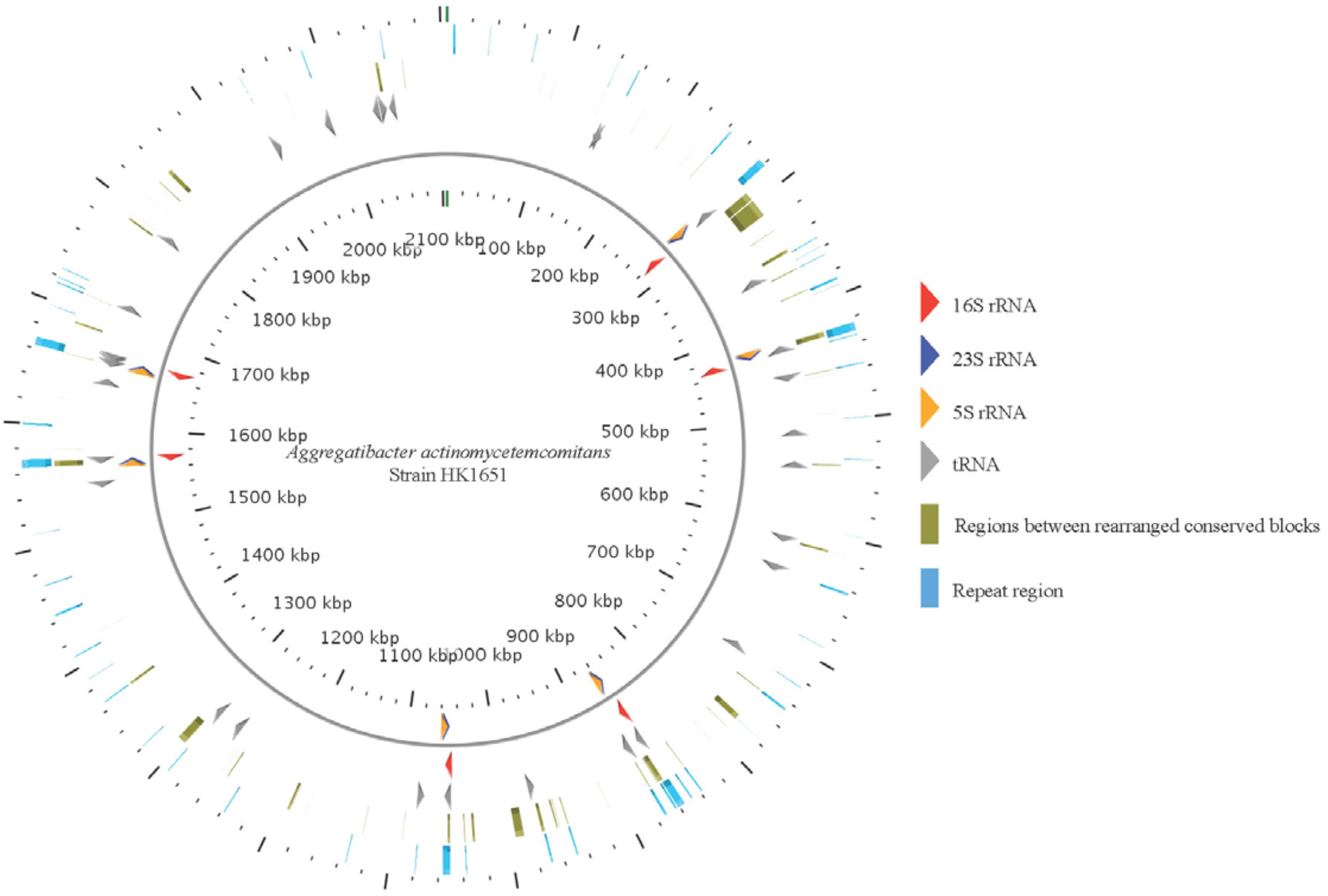

Circular chromosome map of HK1651 genome with the locations of genes of the rRNA operon, the repeat regions and the inter-LCB regions (based on comparison to strain D11S-1).
