# Supplementary material for: Markedly different genome arrangements between serotype a strains and serotypes b or c strains of Aggregatibacter actinomycetemcomitans
Source: BMC Genomics. 2010 Sep 8;11:489. doi: 10.1186/1471-2164-11-489 (PMC2996985; doi:10.1186/1471-2164-11-489)
Supplement: Additional file 5 — PDF Repeat elements in A. actinomycetemcomitans. The FASTA sequences of the repeat elements in A. actinomycetemcomitans strains D7S-1, HK1651 and D11S-1 [file 1471-2164-11-489-S5.PDF]

Additional files

Additional file 5 –Repeat elements in *A. actinomycetemcomitans*

```
>REPEAT01
TACTCTATAAGGACCTAGTACTTCACATGCCCTTCAAAGGGCATATGAAAACCAACAACC
GTGAAATAACTCTATGACCTACCCCTTTAAAGGGGTCCACATATCCTTTCTTCGATAAAT
TATCCTGAATCATGTCTTCCTTTTCCTGATTCCTTATATACTCCTACACTACCTTTGTAT
TTAGCCCTACCGTGCTTACATAATAGCCTTTCGACCAAAAATGTCGGTTACCATAGTTGT
ATTTTAGGTTTGCGTGCCTTTCAAAAATCATTAACGATGATTTTCCCTTTAAATACCCCA
TAAAACTTGATACCGATAATTTCGGCGGAATTTTTAGAAGCATATGAATATGCTCTTTCA
TTGCGTGCGCTTCTATTATTTCCACATTTTTATAGTCGCATAACTGCCTTAATATGCTAC
CTATATCCGTTCTAAGCCTTCCGTAAATGGCTTTTCTTCTATATTTCGGGGTGAAGACGA
TGTGATACTTACAGTTCCATTTGGTATGTGATAGACTTGAATCGTCATTGGCTTTACTTA
CCATTGACTTATCCTCCTATATCTTCAATTTTGGTTGTCAACCTTATTCATTATATAGTG
AGGATTTTTTTATGTTGACCGCTTAAGCTCTTTGATTCCATACGCATAGCGTACGGTTTT
TATAGCTAGACAACGTCTAGCTATAACAAAAAGCCCCGATGCTCTCACATCAGGGCTCAC
TATCAGGCTTGGCGGTGACCTACTCTCACATGGGGATGCCCCACACTACCATCGGCATTA
CGGCCTTTCACTTCTGAGTTCGGTATGGTTTCAGGTGGTACCACCGCACTATCGCCGCCA
ATATTCTTTGATGACTATCCTCTCTCCTTTCCGCGCTTTCGCTTTCTTATTCCGCTCTTC
GCTTCTCTCGTTCTTTCTCTTCTTACTTCCCTTCCCGCGTACTATACGCGTCATCTTCCA
AAAACAAGCCTCAAACCAACTTCCTTCCCTCAACTCCCTCTCCTGAGCTACAATCCTTTT
TCTTCTCTTTATCTTTCTCTTTCTCTTTCTCTTTAATTTAATCTAATACCGTTATTCTTA
TCAATACATCCCCACGCTCCTGCTCCCCCGCAAAAACACTTGAGCGTTGTATGGCTAAGT
CCCTCGGGCAATTAGTACGTGTTAGCTCAACATATCACTACGCTTACACACCACGCCTAT
CTACGTCTTCGTCTTAAACAACCCTTACAGACCTAAAGTCTGGGAGAACTCATCTCTTGG
CAAGTTTCGTGCTTAGATGCTTTCAGCACTTATCTCTCCCGCACTTAGCTACCCGGCTAT
GCGTCTGGCGACACAACCGGAACACCAGCGGTGCGTCCACTCCGGTCCTCTCGTACTAGG
AGCAGCCCCAACCAATTCTCCTACGCCCACGGCAGATAGGGACCGAACTGTCTCACGACG
TTCTAAACCCAGCTCGCGTACCACTTTAAATGGCGAACAGCCATACCCTTGGGACCTACT
TCAGCCCCAGGATGTGATGAGCCGACATCGAGGTGCCAAACACCGCCGTCGATATGAACT
CTTGGGCGGTATCAGCCTGTTATCCCCGGAGTACCTTTTATCCGTTGAGCGATGGCCCTT
CCATGCAGAACCACCGGATCACTATGACCTACTTTCGTACCTGCCCGACCTGTCCGTCTC
GCAGTTAAGCTTGCTTATACCATTGCACTAACCTCACGATGTCCGACCGTGATTAGCAAA
CCTTCGTGCTCCTCCGTTATCCTTTGGGAGGAGACCGCCCCAGTCAAACTACCCACCAGA
CACTGTCCGAACACCTGTAATCGATGTCTCGTTAGAACATCAAACGTTAAAGGGTGGTAT
TTCAACGTCGACTCCATAATGACTGGCGTCACTACTTCATAGTCTCCCACCTATCCTACA
CATCAAAATTCAATGTTCAGTGTCAAGCTATAGTAAAGGTTCACGGGGTCTTTCCGTCTA
GCCGCGGGTACACCGCATCTTCACGGCGATTTCAATTTCACTGAGTCTCGGGTGGAGACA
GCCTGGCCATCATTATGCCATTCGTGCAGGTCGGAACTTACCCGACAAGGAATTTCGCTA
CCTTAGGACCGTTATAGTTACGGCCGCCGTTTACCGGGGCTTCGATCAGATGCTTCTCTT
GCGATTACACCATCAATTAACCTTCCGGCACCGGGCAGGCATCACACCCTATACCTCCAC
TTTCGTGTTTGCAGAGTGCTGTGTTTTTAATAAACAGTTGCAGCCAGCTGGTATCTTCGA
CCGGTTCAACCTTCAGGGGCAAGCCCTTACAATCTACGCCGGCGCACCTTCTCCCGAAGT
TACGGTGCTATTTTGCCTAGTTCCTTCACCCGAGTTCTCTCAAGCGCCTGAGTATTCTCT
ACCTGACCACCTGTGTCGGTTTTCAGTACGGTTTAGTAAAGCCTTTCGCTTAGTGGCTTT
TCCTGGAAGTATGGTATCAGTTACTTCAGCCCCTTAAGGCCTCGTCATCATCTCTCAGTG
TTTATAGGAGCCCGGATTTGCCTAAGCTCCCCACCTACCAACTTAAACGTGCATATCCAA
CAGCACGCTAACCTAACCTGCTCCGTCCCCACATCGCAGCTTTACCAAGTACGGGAATAT
TAACCCGTTTCCCATCGACTACGCTTTTCAGCCTCGCCTTAGGGGCCGACTCACCCTGCC
CCGATTAACGTTGGACAGGAACCCTTGGTCTTCCGGCGAACGGGTTTTTCACCCGTTTTA
TCGTTACTTATGTCAGCATTCGCACTTGTGATACGTCCAGCATACCTCTCAATACACCTT
CTTCCGCTTACACAACGCTCCCCTACCCAACAGGCGTATCACTAATATTCCTCAATTCCC
AACCGCACTTCCCGTCATGACTTCCGCCTCAACGTGTTTGGCGCTTGACCAACTTGCTTC
TCTTCATTTCGCAAATTGTTAATCGTAAGGCGTATTAGTGATACGCCTGATGCCGCAGCT
TCGGTGACTAATTTTAGCCCCGTTACATCTTCCGCGCAGGCCGACTCGACTAGTGAGCTA
TTACGCTTTCTTTAAATGATGGCTGCTTCTAAGCCAACATCCTAGCTGTCTAAGCCTTCC
CACTTCGTTTCCCACTTAATTAGTACTTTGGGACCTTAGCCGGCGGTCTGGGTTGTTTCC
CTCTCCACGACGGACGTTAGCACCCGCCGTGTGTCTCCTGAGTATCACTCTTCGGTATTC
GTAGTTTGCATCGGGTTGGTAAGCCGGGATGGCCCCCTAGCCGAAACAGTGCTCTACCCC
CAAAGGTGTCCGCTCAAGGCTCTACCTAAATAGATTTCGGGGAGAACCAGCTATCTCCCG
GTTTGATTGGCCTTTCACCCCCAGCCACAAGTCATCCGCTAATTTTTCAACATTAGTCGG
TTCGGTCCTCCAGTTAGTGTTACCCAACCTTCAACCTGCCCATGGCTAGATCACCGGGTT
TCGGGTCTATACCTTGCAACTATTCGCCCAGTTAAGACTCGGTTTCCCTTCGGCTCCCCT
ATTCGGTTAACCTCGCTACAAAATATAACTCGCTGACCCATTATACAAAAGGTACGCAGT
```

CACCCTTTCGGGCTCCCACTGCTTGTACGTACAAGGCTTCAGGTTCTATTTCACTCCGGT
CACCCCGGTTCTTTTCGCCTTTCCTTCACAGTACTGGTTCACTATCGGTCAATCAGGAGT
ATTTAGCCTTGGAGGATGGGCCCCCCTTCTTCAAACAGGATATCACGTGTCCCGCCCTAC
TTCTCGTTAGCTTAGTACCACGACCCAGACTTCGAGTACGGGGCTATCACCCTGTCTCGC
TGTGCTTCCCAGCACATTCCTCTGTCTCTGTCGCTATCACTAACAGGCTCTTTCGCTTTC
GCTCGCCGCTACTCACAAAATCTCGGTTGATTTCTTTTCCTCGGGGTACTTAGATGTTTC
AGTTCTCCCGGTTCGCCTCATTACCCTATGGATTCAGGTAATGATAGTGGGTTCTTCACC
CACTGGGTTTCCCCATTCGGATATCTCGGATTAAACGCCTCTTATCGACTCATCCGAGCT
TTTCGCAGATTAGCACGTCCTTCTTCGCCTCTGATTGCCAAGGCATCCACCTTGTACGCT
TTGTCTCTTAACCATACAACCTCAAGTATTCTTGCCCTCTCATAGGATATTAGTATATTC
ACCATAAGTGGCATACCCGCGCTGCGATTAATAACAGTTTTAAATTAAAGTCGAGTAATC
GCCTTTTCTCAATCACTCGCTCAGACTTCCTTGAAAGTCTCGTTTTCAGCTTGTTTCCGG
ATTTTTAAAGAACAGATAAGAATAATGTAAGTCTATAGACTCACTATCATCATAGTTAAA
TTCATTCATCGAAATTAAAAGATACCTTAAATAAACTTAACTATGACGATGTTGGTGGAG
ATAAGCGGGATCGAACCGCTGACCTCCTGCGTGCAAGGCAGGCGCTCTCCCAGCTGAGCT
ATATCCCCAATCATATCCTTTGGCTTTCACTCATACTCAATGCGCTTGATGTATAAGTGG
TGGGTCTGAGTGGACTTGAACCACCGACCTCACCCTTATCAGGGGTGCGCTCTAACCACC
TGAGCTACAGACCCAACAGGATTTTGTCTTCGATGTCGTTCTTCCATTGCCTACAATCAT
CAAACAAACTGTGTGAACACTTAAAGTCGTCTATTGGTAAGGAGGTGATCCAACCGCAGG
TTCCCCTACGGTTACCTTGTTACGACTTCACCCCAGTCATGAATCATACCGTGGTAAACG
CCCCCCTCTCGGTTAAGCTATCTACTTCTGGTACAACCCACTCCCATGGTGTGACGGGCG
GTGTGTACAAGGCCCGGGAACGTATTCACCGCAACATTCTGATTCGCGATTACTAGCGAT
TCCGACTTCATGGAGTCGAGTTGCAGACTCCAATCCGGACTTAGACGTACTTTCTGAGAT
TCACTCCCCATCGCTGGTTGGTTACCCTCTGTATACGCCATTGTAGCACGTGTGTAGCCC
TACTCGTAAGGGCCATGATGACTTGACGTCATCCCCACCTTCCTCCGGTTTATCACCGGC
AGTCTCCTTTGAGTTCCCGACCAAGTCGCTGGCAACAAAGGATAAGGGTTGCGCTCGTTG
CGGGACTTAACCCAACATTTCACAACACGAGCTGACGACAGCCATGCAGCACCTGTCTCA
AAGCTCCCTAAGGCACAAACCCATCTCTGAGTTCTTCTTCGGATGTCAAGAGTAGGTAAG
GTTCTTCGCGTTGCATCGAATTAAACCACATGCTCCACCGCTTGTGCGGGCCCCCGTCAA
TTCATTTGAGTTTTAACCTTGCGGCCGTACTCCCCAGGCGGTCGATTTATCACGTTAGCT
TCGGGCACCAGGGCTAAACCCCAATCCCCAAATCGACACCGTTTACAGCGTGGACTACCA
GGGTATCTAATCCTGTTTGCTCCCCACGCTTTCGCACATCAGCGTCAGTACATCCCCAAG
GGGCTGCCTTCGCCTTCGGTATTCCTCCACATCTCTACGCATTTCACCGCTACACGTGGA
ATTCTACCCCTCCCTAAAGTACTCCAGACCCCCAGTATGAAATGCAATTCCCAGGTTAAG
CCCGGGGATTTCACACCTCACTTAAAGGTCCGCCTACGTGCCCTTTACGCCCAGTTATTC
CGATTAACGCTCGCACCCCCCGTATTACCGCGGCTGCTGGCACGGAGTTAGCCGGTGCTT
CTTCTGTATTTAACGTCAATTTGGCATGCTATTAACACACCAACCTTCCTCATCACCGAA
AGAACTTTACAACCCGAAGGCCTTCTTCATTCACGCGGCATGGCTGCGTCAGGGTTGCCC
CCATTGCGCAATATTCCCCACTGCTGCCTCCCGTAGGAGTCCGGGCCGTGTCTCAGTCCC
GGTGTGGCTGGCCATCCTCTCAGACCAGCTAGCGATCGTCGGCTTGGTAGGCCTTTACCC
CACCAACTACCTAATCACACTTGGGCTCATCCTATGGCATGTGGCCCGAAGGTCCCACAC
TTTCATCTCCCGATTCTACGCGGTATTAGCGACAGTTTCCCGTCGTTATCCCCCTCCATA
AGACAGATTCCCAAGCATTACTCACCCGTCCGCCACTCGTCAGCATAAGTACAAGTACTT
ACCTGCTACCGTCCGACTTGCATGTGTTAAGCCTGCCGCCAGCGTTCAATCTGAGCCATG
ATCAAACTCTTCAATTCAAAAAGCTTAATCCCTCAATAGCTGACTTAATTAATCTATTAT
AAATAATATAAATAAACGAATCTTCCGGCACCTATTAAGTTCAATCTTAAAATTATCTAA
AACGAATCTATAAGTGCCCACACAGTTTGTATGATTGATTGTTAAAGAACAAAAAACGAC
GCGCTGTCTATAAAAACCTACAACGGCGCGTCGTTGTGCCGGCGTATTATAGGCTTTTAT
CCTTACCTTGCAAGTACTTTTTTAC

>REPEAT02
GgAAAAGTGATCTGCCCCCcAAAAGTTGGACAGGTTAGTTAACTTAAaTATTGAGCTCGG
TATtGcACCGGGCTtAATCcTTTTAAATCAAGTTTAATTCGTTCTTGGTTGTAATACACC
AAATATTCTTCAATTTCCGCCTGTAATTCGGCAATCGAAGTATAAGTGCGTGAGTAAAAA
CACTCAGATTTTAATATCGCAAAAAAGCTTTCAATCACCGCATTATCATAGCAATTCCCT
CGGCGACTCATACTTTGTATCGCCTTGCCTTCCAACATCTTTACCCATTCTGCTGAGCCG
TACAATACCCCTTGGTCGCTGTGAATAATCGGGCATTCGCTCGGTTTTAGTTTGCCAAGT
CCTTCTTCCGGCATTCTTTTTACCAATGAGAACTTAGGGCGTTTCGCAAAATTATAGGCA
ATAATTTCCCGGTTCGCTAAATCCATCAACGGTGAAAAATAGAGCTTTTCTTCCCCAACT
CGAAACTCAGTGACATCGGTTACCCATTTTTGATTGAGCGCCGTCGCTGTAAAATCACGA
TTTAGCACATTCGGGGCAATATGCGATGTTTTCCTCGTTTTCCATGTCTTTTCTTGCGTA
AAATAGAATGAATACCTAACGCATTCATCAGTTTTAACACCGTTTTATGATTCAAATGGA
AACCCATTTAaCGTAaTTTGAGTGTCATTGGGCGATAGCCATCTCGTTTTCTGTTCCTTT
TATACCGCAACAAGATAGTTTCTTTTATCTCACGATAATCGTGTTTAACTTGCTGATAAT
AAAAGATAGAACGAGGCATTTTAGCCACACGAAGTAAGTCATTCAACGCGTGATTTGGCT
TCAATCTTTCAATGATTTTtCTTTTTTCTGTCGCTGTTTTTGACGGTCGAGTGCCTCCAA
CTCCTTTAGGTAAGCAATCTGTGCACGTGCTAATGCCAGCTCTCGTTGCAGCTTTTTAAA
CGCTTTTGGGGAAAAGTCTGTTTGTTCCGGGATTTCAATCGCTTTCTTTTTCATCTTTGG
CTTCACTATTTTAGGGCGTTGAGGGTTTATATAAGGGGATTTTACGCCATtAAGCCCTCT
TTCACGAAAGGCTTTTCCAATAAATGACTTGAGAACGAGAAATCTGATGAAATTTAGCCA
CCTCACGGATACCAAAATCCTGCTCGGTGACCTGTTTGATAGTTTTTtAaCGAAATTgaT
AAGAGTAAGACATAATCTGCACCTCAAATTAGGTGTCCAGGTTTTGGGGTGCAGAGCAAA

GTGCGGTCA

>REPEAT03
AATAACAATCCGCACGTAAAACGTGCGGTTTTTAGGCTACccTATAAGGgCCTAGTACTT
CACATGCCCTTCAAAGGGCATATGAAAACCAACAACCGTGAAATAACTCTATGACCTACC
CCTTAAAGGGGTCCACATATCCTTTCTTCGATAAATTATCCTGAATCATGTCTTCCTTTT
CCTGATTCCTTATATACTCCTACACTACCTTTGTATTTAGCCCTACCGTGCTTACATAAT
AGCCTTTCGACCAAAAATGTCGGTTACCATAGTTGTATTTAGGTTTGCGTGCCTTTCAA
ATGATTTTCCCTTTAAATACCCCATAAAACTTGATACCGATAATTTCGGCGGAATTTTTA
GAAGCATATGAATATGCTCTTTCATTGCGTGCGCTTCTATTATTTCCACATTTTTATAGT
CGCATAACTGCCTTAATATGCTACCTATATCCGTTCTAAGCCTTCCGTAAATGGCTTTTC
TTCTATATTTCGGGgTGAagACgaTGTgaTACTTACAGTTCCATTTGGTgTGTGATAGAC
TTGAATCGTCATTGGCTTTACTTACCATTGACTTATCCTCCTATAtcTTGAATTtGGgTT
GTCagCCTTATTCTTATATAGTGAGGATTTTTTATGTTGACCGCTTAAGCTCTTTGATTC
CATACGCATAGCGTACGGTTTTGTTTATAGCTAGACGTTGTCTAGCTATAATAAAAA

>REPEAT04
ATCAATCTCCTCTTTTGCAAGGGCAGGTTCCCGTTTCAGTTTTTAAAGGCTTGTGGTGA
AAAGTCGCTTGTTTCAaGCCACTTCAATCTGCGTTTTTTTCATTTTGGGTTTCAGAATTT
TGGAGGTTTTAGGTTTGTG

>REPEAT05
TAAAGAAAAAACAAAAGTGCAGTCATTTTTCCAAATGTTTTTTACTCCCACAAAAACACT
TAAAATTTTAACCGCACTTTTAAATAAGAGATAACTACTTTCTATTCTTTAATTTCAATT
TCCGACTTTTCCATTTACCCGGTGGCTTAGGAAATTGCTCCATATCTCCCGCCTCATTCT
CCCAGGCGATACTGAATCGATTGTAATGTAATGAACGCATAGGATATTCATATAAATTCG
GACAAGGCATGATGCCATAACCCGCTTTGATATTATCTTCTACCGAAATAATCTTCATAT
TCTTAAATTTCCATTTGAGTTGCATACCTTCTTCATAAGTGCCCATCTCAAATGAAGGAA
GTGCTTTAATATGAGGTGCCTGTTCGACAAAATATTTTTCAATCTCTTTTTCTAACACCC
CGTCTTTCGGCAATTTACGGCAATATAAGCCGCCATCTATCCAACCGAATAGAACTTTCC
CCAAACATTCAAAAATACCGGGACGGGTTTCCAGCGCTGTCATCATACTTGCAATGCGTT
CCGGCGGTGTATCGGGATTCATAAAATCCACTAAAAAAAGCTTCAATGTGGCTGCCGAAC
TTTTTTTATACAACTTTGGATTTAACGTAAATATCATACCTAACATCGGTAACCACATAC
TGTAATCCGAGACTATCATTTGTGAACCTTACCGAAAGATTTTAATCTTGTGGGGATACT
CATAAGGGTGTACCCAAAAGACCACCGCGCCCCCTGTATTGATTAGATATTCCGGAAACG
GGCGCATCAGTTTATTCACTTCCGTTAAATAAAACTCTTTATTCACGTAAGTGTAAATAA
TCCCGCGCTTGCGGTCAAAACGTACTCGGCGTTTGGTTGGCCAGAGGCAATATATGAGAA
TAAGAACATATTTGCCGACAGTAGAATACCCCCACCATCTTTCTTCTTTGCGGGTTTTAT
CAGCCTCAAGTATATATACTTCTTTCGGTTCTCCCCAGCGAGTAACATTAGGGTTTGAAG
TGGATGCTACTTTAGAATATTCCCTTTCAGCCCACACTTCGGGTTGAAACGCAGCATAAA
TATGAGTTTTTGTCATATTTTGGTAACGAGGATCAATAAAATCATAGTAAACAGCAATTA
CTGCAAATATAATGAAGACAATTTTCACTGCCGCCCAACGCTTTTCCTTTAACTCAATGG
TATTTTCACCGGTTTTTTTAAACCAGCGACGCATTAAACGTTGCTCTTTCTTTGTTAACT
CACTTTGCATAACCTACTCCGAATAGCCTTCATCTCTTGCATCTTCTACTGCTTTCTGCA
TACGTTGTTCAAGTTCACGCGTTCTGTGATTAAATTCCTCACTCAAATACACATCTGTGA
ATGTATCACCTTCTTTATCCGTGAACTCGGCAACAATGCGGATATCTTCTATATGGATAT
TATTCTCGGCAAGATAAAATGTCACTTCACCGGAAGCAGCGGCATAAAACCGTTTAATCG
GTTGTGCTTGAGCCAAACCCCATTGATAAAGGTTAATCACCAAATTCTGTGTTGTCGGTT
CCGTGCGTCTGAATTCTGCGCAGCTAATACGATAATAGTCTCTGCTGATTTGTTGATTT
TTAATCCCGATTGAATATCATATTCATAGAGTTCTTTTTCAAAGCCTTGACTGATTAAAA
TATAGTCTTCAACCTGCTCATTCATAGGCAGTCCAGCACCTGCGACAGCATCTACCTGCC
CCGTCGTTACCAATTCCAATCTTGCAAGATATTGTTTAGCGATTTCTGCTATGTTTTCAT
TAGCTAAAATTTCTGCGCGCTTAATTTGATTATTAACATCATCGCGTTTTTCATTCCAAT
AAAAATATGCTTTTCTTCCCCAAAATCCTTTCTTAACCCATGTCTCTAATGGTGTTGAAC
CAAATAACGCTATCGCCGCAAAGCCAAGTAGAACAATCACCAACGCATACGGGTTTGCAA
CACCTAACGTTGAAGCCAATGTTCCCTTACTTACCATCGCGACTATTCCGGACAAGACAA
GAACGCTTGAGGAAGCGATAATCGCCCCATTTGCCACAATCGAAACATTATCCTCATTGA
CAATCGCCTCTTGTAGTCCGCCAAACGCAATCACCGCCGTCAACACGCCGCCGTCAACCA
CCATGCCCGTATAAGCCAACGCCCGCACACCAACTAATACCGAACGTGCCGATGCACCGG
CAAGATTTCTTAACGCGCCTATACCCGCCCCCGGCTTACCCATCAGCCCCAGCGCATACG
CCGTATCCGCCGTCTTCACCATCACCGGGTCTAATGCCAATCTTCCCGCCGCCGTACGTA
CAAGTTTTCTTGGCACCGTTAAATCATTATTCAAGGACAACATTCCTGCATAAATCGCCA
ATACCCCAGTAAAGCCATAATAGCGTTCGCTAAGCTGTATGCGGGCTTCCAAATCCCTAA
TTTCATCAAGCGTGATTCTATTCTGTTCTTTTAGCGTTTGCTTGATTTGTTTTAATTCCT
GTCGAATTGCTTTCTTACCTCTACCAGTTTCATGGCGTTCTTTTTTATATTGTCTCGAAC
TGTCTTGTTTCATCAGCCGGTCTAAAAACTTGTCCGCCACTTCGGCAAAGAAATAATCGG
CATTTTTTACCAGCCTGGCTTCATCCCAGCCATAAATTGCCATCACCTTGTCAAATTGCG
TTTTTCTTAATGCCACTAAATTTGCCATTGTACGACCGCTGTTTAAATACACGTGCTCCA
TGGCTTGGAAATAGCCTTTTAACAGATTCTGACGCTGACGCGCCGGGATTTTCATCGCCA
CTCCGTACACCGCATCGGTAACCTTCTTACTATGCTCCTTCAGCCAACCGACTGTCGTCG
GTACCTTACCGGCAGGCGCCGCGTGCTCACTGAATAAACTTTCCAGGACCGCAATGCCGT
CAGACGAACATTCTAACCCATAGGTAAAATCTGCCAGCAGCCGATAAAACACATAAAAAC

CACGGTCTATACAACCCTCTTTGGGGACATAACGACCAATATCGCCGGAAACCTTAAAAA
ACTCCCGGAATGACGCACGTTGATTTGCCAGGTTCGTTACTTGTTTTACTAACGCATTTA
TCGGCTTTTCATAACGCTCAAACGGCACGTTCAGCAACGCCGTCATTTTGCTGTTAAATT
CGCTAATCAACGCCCCCTGCACGCCTTTTTGTGCAATCACCCGTTCTTTTTCCTTAAACG
GATAAGTCTTCCCCCGTACATCAAAACCAATTTTCGGGTCGATAATATTGCCGATAGCTA
TCGGATAAGCATAGCGTGCATAATAGGCTTTTTGTTTTTCGGTTAAATCAATAATGATTT
TCTGTAATTCATGCATTTCGCCAACCACATCCTGCAATGCAACCATCAAACCTTTTTCTT
CGTAGTCGGGTAGCCCCGTTAAAGGAGAGGGAATCGCTGCACTGTTTAGTTTTGTATACC
AGTCCTGAGTTTTATCGGCAAAATCATCAGATTGATTGCCGGATTGGTATTCTAAAACAT
GAGTACCGAGTGATTTCAGCGGGGCTGAATGATTCTCCGGAGCGTCTGTTCTGACAGTAG
TCATTATAGCCTGTCTCAATGCTGCCTTTGTCTCTAACAAATGAATGATTTCCGATGACC
ACATATACTCACTATAAGCAATGTCAACAATATCTACGTCTTTATCAACAAATGCAGTAT
TGAAACTTTTCAGATGTGAAGCACAAGGATAGTTTTTCGTATCAATTTGTCCAAGAGTCC
AGTTTCCATTTGCACCCTCCTCACCCCATTTACAAAGTAAAAAACAGGCGTTTAATCCTG
CATTTTTACCATGGCTCAATTGTTCAAAATCACTTGCACTGTTTGCGTCATCATTATTGG
TGACATAGCGAAAAACAAGCCATTTGCCTTTATCATCGGTAGAAAACAGGCGGTGCTTAG
TATGTGCATAGATATAAACAAAACCTTGACGCAGTTGCATAAGTTCATAGTCGGGAGAAG
CACCGATAGTTCGGGGCGCCCGGTCAGGCAGGTTTTTCACTGTTCCATCACGGGCATCAG
CTAAATAACCAAGACTTAATCCATAGCGAACAGGGTAAAGCGGGATAACCGGTAATTCGC
ATTTGCTATGCCCATCAGATAATTCTTGCGCTTTTTGATTGATTTTATCCAAGTCGAGCT
TTATTTTTGTTTTTCGTTTTGTTTGAGTGTTAGCTGTCATTGTTATTATTTTCTATGTCA
ATTAAATGATTCATTAATGTTTCAAGTAGGTTTGTATTTGAATAATGCGTTTTTTGCCAC
CATTGTTCCAGTAACTTAAAATAGTCCCACTCATTATTTTCTGCAATTAAACGACCAATT
AAGTAAAACCAGTATTCGCGCTGAGATTGGGCTATATTACGTGATTTTGCTTCCTCTAAC
CATTTGCCGATAATATCTGAATCGACATTATCAATATGTTCTGTAGCCATGCTTTTTAAC
TTAACTTTTGTATTAAGCCATTTTTCGTAGGCGAATGCCTGTTTCTCAACTTTACCCAAC
TCAATCTTGGCCGGCAGCGTATTGTCCGCTAACGGATTTAACCTAAAGCTGATAAATTCC
TCCTCCACACGTAAGCCAAAAGCCTCTATGATTTGCTCCTGTTCATAAGATTTAGCGGGT
GCTGCAGAGGCTTCGGAAAGTAGTGTTTTCACCCCGAACAGGGCCGCCAGTTGTGCAGGA
TAATGGCTGAGTTCAGACAGATAACGATTAAGTACCAAGGGGTCATAAAATCGGAAGAAA
TACCATTTCTCCCTATCAGGGTCATAAAGATGTGTCCATTTGCGCAGATGACGATAAACC
TCATCAAAGCCTTTGCGACTTTTAAGGAAAATCACTGGATTAATTGCCCAATGACTGAAC
ACCTTGCTTTCGCCCCGACGAAACAGACGCTGCAAAAAAGCCACTGTCTCTTGTTGATAC
GGGTCAAATTCCAGAAGATACGGTGCCTGCTCTTCCAGCGCTTGACCAAGACTGCCAGTA
TAGAGATTTTCCAGGCGTCCCGCTGTTTTTAAGCGCGATGGAAAACTCACCGCCTTGGCA
CAGTCCACCACTGCATAAAAGTGCGGTGGCCTTTGCACTTGGTTTTCGCGTTCTCTTTTA
TGTTCATCTTGATTCAGGATTTTATGATATTCCCCCAAACCCTCAAAATCCGCGAAAAAG
AATGTCCGCACTTCCCGTGGCATCACTTCAGCAACAATAAATTCCGTAAACGGCTCAAAA
TGCACTGATTGTTGATTGAGATACGTTTCAAAATGCTGTCCGTGGTTTTCGGCTTCAGCT
TCGGTGTATAGGCACAGCAGTGGCGTCTCGGTGGTTATTTGCTGTGCCGCACGCCAAAGC
TTTGCCTCAAAACCATGGCGGCTCAGCCAACTCTGAATCGGCAGCGGGGCAAGCTGTGCC
TGTCCTGTCAGACCCTGTTCTGCCAGGTAGGTATTGATTTGACGCTGATAATCCTGCGTA
TCTGCGGCAAGGCAAAGTAAATACGCCGTGCGGGTGGAAGCAGGCTTATCCTGCCTGCTC
AGATGATAATGGGTCAGTCGGATTTGGGGATAGGGCGCTGTTGTGATGTTATCAATGTGC
ATAATGAATGATGATGGAAATAAGGGAAATTAAGGAGATTGTTGACTTAACGTTGACAGG
CACATTGTATATAACAATCTGACTTTCAGCAACAGCGCAAAGACAGTCGGATAAACCGAT
TAAAAGTTGCAGAAGGAAAACCTCATGACTAAACTGTTTAGCCCGGTATTCAAAGCAGTA
TCTGAAAGATAATAAGGTATTTCGTCAAACAAATCGGGCGTTGTTTTGACATTCATCGTT
CAGCCCCTTTGTGTTAGAAGAAGTTGAGCGTTATGTCAGATAGGATGGGAAATCGGGCGC
AATAAAACCCGTAAGCTGATGACAGCATTACAACTGAAGCCCCGTTATACACGTCCGACG
TTCTACCGCAAACCGGTCAAAACGTTTGCGGATAATTGGTTAAATCAGCAATTTAATGTG
CAGGCGTCAGGTATGGCCAGGTGACATGACCTATGCGGTAACGGAGCGGTTTTTCCGCAG
TTTAAAGACGGAGAAACTTTATCGGGAGTATGCAACTAAAACTGCCGCACTGCTTGGTAT
TGCGGAGTATATTGAGGATTTTTATAATTCGAAACGGCTTCATTCGGCATTGGGTAACTT
ATCACCGATGATATTTGAAGCGAAACAACCGTGTTTAACACTTTAAATATATCTTTGCTA
TGTTGCGGAATTTTGAACCATTACAT

>REPEAT06
AAAAACACTCAAAAAACCAACCGCACTTTAGCACTAATACGCCACCCAGAATTTTTCCAG
TTCCATTTTATCGGGGTGATAGTGATAAACATTGATGCCGCCGTGATGCGCCTGTCCGGT
CACCAGAATATGCGCTTTCGGGGCAGAAGCGACGCTGTTTGATTTTTCATAGGCATCTGT
TGAAATAGCAGGATCGTCACCAATTTCCACAAAGGCCTTTATCCACGATCGCCCACCGTA
AACAGCGGGCTGTGCTTTTCCCCTTTGCCATCTCTTGGCGGCGCCATTCTTAATTCAATA
AATTCCGTGTCTGTTACAAAAGCATAAGTCACCGGATTATTTAAGTCGATCTCTACACCC
GGCACGCGCAGAAGTTGACTATCATCATATCCTTCAGAATTTCGAATTCCTCGATCAGGG
TCAGCTAATTTCAATCGGTTTGTGAGATTAGCGGTTTCCCACATATAGTGGCGTATATTT
TCTCCGCCACCGACGATCCATTTTCCGTCCGGACTGAATAACGCATCAGAGCGTCCGCCG
AATCCCCAAAGGCGTGCCGCCGGTTTCAAGGTTTCTCTGTCCCATAAAGTCACACCATCA
TAGCTACTTTTTTTATAAACGCTGGGTTGCACCGGATCGGCGGTAGGATTTAATTGCACA
ACAGCGCCGTCTTCTCTGTAACCGCTACCTGATGAAGCAGTCAAAAAATATTTATCGGAT
AAGTCAAAATTATAATGTCGCCAAAGTGGGGTATCGGTATAAATCGGCACCATATTCTCA
CCATGCCCTTTAAAAATCTTGCCGGTTTGGTTGGCGCTGGCATAAAAGCTTCGGTCGGCA

CTCATGATGTGTCTTGTTAAGTCAAAATGCTTAAAACTTTCCAGCTCTTTTCCCTCCACA
TTTTGAATATGCACGACATTATCCAGCCCCTGCCACATAAAATCATGGCTGTCGGGAATA
AAATAAGCACTGTCTGTATTCACATCTTCAGCAATCACTTTTTTTTCCCTTTTGTCGATA
TTCCATAAAACCAGCTTAGTCTTATCTTTCCTTTTATCATTTTTCCAACGGTGGGCACTA
ATCACATATTTCCCATCACCGGAAATTCCCAATGTTTGCACTTCTCCCGTTTCAGATAGT
AAATCCTGCCAGTGGGTAAACACATAAAATATTGCATAACAAATCAAGGCAAATACGCCG
GCAATAAAGAGTTTCTTCTTCATGTTCATAGCTAATACCGCCCGAATTTTGAATATAATT
TTATTTTTTTATCCATAATTTCCCGCTTATAGCTATATTTATAAATCAACCGGAAAACAT
CATGCACATCAATCTGTGGATATTCCCACTCACGCATGGCTGAATGCGAAATAAAGTAGT
AAAAATATTCACCATTCCGAATTTCCCACTGCCCAAAGAACTCAAGCTTATCTTCTCCTT
GGTTAAGTAGCTTTTCAATAAAGTTATCATGCCAACGCTTTTTCTCTTTTTTGGAAACTT
CCTCACCCCATTTATCCTGACCCAATGTCGTTCGTCTGTTTCCGCAATGCCCCAAAGCCC
CTCTCACATCCGAGCCCTCGCCGCTCCAGTTCGACAGTACATTATCAGTCAGCACTTCTA
TAATATGCTCCAACACCTCCCCACCGATAGGAAAACGTCTGAAATGGCTCCAGGGTTTTA
GATAGTTCAGCTCGGCAATCGCGCTATGGTCGTTGACTTGCTTAGCTTCCCGTTCAAGCA
ATTCCCGTATAGTTTGACGGCAATCAGTTGTAGAACGAGGTAATTCACGCTGAATTTGAT
AAGGGTTATCATAAAGTACAGTCTCATAAATCGCCTTACGAAATTTATCAATTTTGGTAT
CTTTATAATAGTCACCAAACGCCCATTTACTCTGTCCGATAGCCCAATATTTCTTCGGAT
AAGCTCCACCCAATAAGCCTAACACCTCGTCATCCCGCCGGCTTCTATCTCCATCAAGCA
CCCCGTCCTCCTGCGAATACAACACGCTAATGTGTTTCGGCACTTTCCAGGCATAAGGAA
AAATTTCCATCGCAATCGGATTAAAGGCTTTCTTCGCTTCATAATTATCCAAGTTGGTTA
AGGCGTTATCCGCCACCGCCGCCTCCCACATAATCAGATTATCCACTTTGTCATTATAGC
CATCGTCAAAATCACCCAACACATTCAGCGCTGAAAGTGCCACCCTGGCGCCTAACGAAT
GGGTAATAATATTAATCCGAATGCCCCTATCAATAAGCGTTTTCAATACCCCTGCCAGGC
GTCTGCCCGATTCATTGGCATACATCTCCGCACGAAAAAAGTTCATATCCGGGTCAACAC
TGCCCGACCACGTCACACCCACAATGCGGCTGTATTTATCCCATAAGTCAAAATCGCTTA
ATTCATCCTTACCTGCTGCCGCAAGATTTAAATAGTATTCCACATTCGGCAACCAGGCCA
GCGCCTTAAAACCATTGTAAGTGTCTTCCAGTTTCTCATAATGTTCGGCTAACTCAAACT
TCGCATAAAAAGGCCGCTCCCCGAGTGCTTTCGGGTATTTTGCATAAGGCTTTCCGTTGA
TTATCTCTATCACCTCTTTCCGGTTTTGTGAAATCACATCTGAAAGCTGTGTCATAAGCT
TGCCGTGCTGATGTAAGAACGGTCTTTGCATCTCTTCGGGATTCAAACGATAGCGATATT
GAACCCGTTCACCTAATTCTTCATCGGTGGCAAAACGTCCCATATGCCCGAGCGGCACAC
TGTAGCCATGGATAAATAATGTGGCATGTCCACCATCATCAATGAGCTGTTGAATAACAC
GTTCAGTTAATTGAACTGCAGGTTCATTTTGGCGCAGACGCAAATTCACCAGAATCGGCG
GCGGTAAATACACCGCCTCAACCTCATTTTCGCTAAAGCTCACCTGCACTTTTAAAGGAT
TGGCAAGTTGTTTCAACGGCTCTTGTGATTGACTAAACTCATCTTCACCATCCGATATCC
GATTCCGCGCTTTTATTAACACATAACCATCCGAGGCCTTAAAAGGCGTGTGTTCGCTCT
CTTCCGTTAAACGGCGGTTATCCTTTTTGCTATAAGTGGAAGACACCGCCGCACCACCGC
AAAGCTGCCAGCAAACCTCACCGCAACGTACACCAAGGTGGACACTCTTTCCACCTTCAA
TTCTACTTCTATAAATCTGCCCGGCTTCATCAAACACCGTCACCTCTATGCCATCAGCAA
GCCAACGGGGTTTATCGGCTGTCTTTAAGGTGTCATGATGGTAAATAGTGACATTCGCTC
GACACATATCTGTTGGACGGTCGATTTGTTCTGAACGATGATTCTCCATCATCTTATTTA
GTACGGATTCTACACTAGACACCGGTTGCTCCTGTTGATTGTAACTGATGATGAATACGC
CCTTGTTTTTCTAATTCATCAAAGCGCTTGTCCAGATAAAACTGCTTCTCCTGTTCGGTT
AAATCATGACTGAGTAAAATCCTGTTCAATTGATTTTCCGGGTCAAGATAATTTACCGAA
TCTCCATACATCAGATTGGCTAGTGCCAGTTGCAGAATACTGCCGCTTTCTCTCATGTTT
GCCGCTTTCACTAAGTGATACGCATGATTCACCGTCTTTTCAACAATACCTTGGCCATAA
GCCTGCGCAAATTCAGGATGTTGCGCCATCAATTCATCCAAGATTTTTTTACACTGGAAA
CAATTATGTTGCTCAATAAACGCCTCAATCTCAAACCTATCCAGCTGCTTTTTCGCTTGT
CTTACCGCTGAAAAATCAACCATCGGTGTATATTCAACCAACTCATCACCACCTTTAATA
CTGAATATTTTCTCAATCATATCGCCGTTAAAAAATGCTGACAGCTTTGTCGGGTAGGTT
TGCAGGCGGTCGAGGTATTCTTCCAAAATAACAGGGTCGAAAAAACGCAAGTAATACCAA
TGCCCCGTATAATCCTGCAACATCAACCATTGCCGAAAATGATTCAATAAGGCATCAAAA
TCCGCCTGTGAGCGAAAAAAGATACCAATATTGCGTTCGCCGTAATTAATACCATCTACA
TAGTTTTCCCCATTGCGGCAAAATAAACGCGGCAGAATATCATGTCCCTTAGGAAGTTCC
ACTAAATAAGGTGCACTACCCGCATAAATCTCCGCCTTTTCTCCTTGAAACAAACAGGCA
TAACGAACCCCCTCTATACGAAACAAATCCGGAAAAAAGCGCGAGGTCGCCGAATTAATC
ACTGCATAGCATTTATCATCAGGGTCATCATGCCATAAGCAAGATTCAATAACCG

>REPEAT07
GNATTTATCGCCCACTGGGAAAGCTTAAAAAATTGCGGACGGGCAGAAGGACCGATTCAT
TGGTTTACGGTGACCAATTCATCGGATGATGGTATCGGGAAAGGGACTTATACCACCAAT
GAAATGTTTAAGTTATTGGGCAGGCAGGAGGTGATGCCGAATGTGGCGCGAGAGATTCGG
CAACAAGAGCTGGAGTTTGAGTTAGGCAAACGCAAAGTTAATCAAGCGGTGTTGTTTAAT
CGTATCATGACCTTGCCTACGGCGGAGGGTAAGGCGCATGCCCAACGGGAATACCGGGCC
AATGAGCAGGAAATCACACGTCTTGAGAAAGAGATTGCCGATGTCAAAGCCAAACAACGT
GAAGTGGAGAAACGGCAGGAAGTGCAACGCACTGAAGGCAGCGGAGATAAAGAAAACAAG
GAAAATAACAAAAACGACGAAACCAACAGCGAATTGAATACTATTGAAAACCGGGAATTG
AGTAAAGAAGAGACCAATCTGACCAAAGCGTTGGGATATGAATTCAGTGATACGCCGGGG
ATTAAAGCGCATATTTGCACCTTAATGCCGAAAGATGAAAACGGCAACTATGTGCATCGT
TACAAAGATAAAAAGGGCAAAGAGCTACCGGCGGAGGTATATGTGCATTCGAAAGTGACC
ATTATGGACGATGTGTTTACCGTTATCAGCTCGGCCAACCTGAACACCCGTAGTATGCAG

GTGGATACGGAATTGGGGATTATTATGGAATGCAACGAGGTGGCGGAAGGGCTGCGTAAA
CGCTTATGGGATTTACACACCAATAAGAACGGGGCGGCAAATCCGGATGATATGCATGAT
TATGAGGTGGCAAAAAGGGCATTTAATATGTGGAAAGATTTGCTTGAATCAAATAAAAGA
GCTCAAAATGATGGGACTTTTCCAGATTGTGCATTACGGGAATTTTATCGGGCAGACCCC
ACAGTAAGTCGGAGTGACTAATGGTAGCGCGTATTTTTAAAACGATAGTGATTGTTATGA
TTGCCATAATCGGATTAATAATTTGGGCAGGTAACAGTTTATTCAAGGGTATTAATTTGG
GCGGCGCAGGTCATTCCGGCGCGCCCGGTATGATTGATGAATATAAAGCGGGAAAAACTCA
ACATGGACAAGATGGAACAGCTGCAGGCGAAGTTAGCCTTTACCTGCAAACACGAAGAAA
AGCCGGAACTCTCCCAAGAAACCCAGCAGCTCTACAACTACGCGCTGTATCATGACCTGC
ACAATATGTGGACAGGTAAAAAAGGGGATGCAATATGGAACGGATTGGCACGCTATTACC
GCATTGCGGCAATGAACGGTGATTACAAGGCGAATATTCGTTTGCAATACCTGCTCAAAA
GCGGACGCATTAGTTCTGATATGCCACAAACAGAAGTGCATAATCTGAATGAAGCGTTGG
CAAAACAGTTGCCGGCGACGGCTTACTATAACTTATATGGTTATTTGGATGTGGGCTATG
GGGTGCGTACGGAGAAGGACGGCAAATATGCTTATCTGAGAAAAGCGGCGGATTTAGGTA
GTCGGGAAGCGCAGTATGTTGTTGGGGATATACTGACGGACATCGATGATGAAGAAACCA
GACCGTTACGCGTGAAAATTTATGAGCAATTATTATCCTGTGCTTCTGCTCAAGGATTGG
GTAAAGCTTCGGTTATGCTAGGTATAGGTCTTCAACGAAAGAATGAATATCAACAAGCAT
TAGAAGTTTTTCATCAGGGTACAAAAAATGGTAATGATTCTTCGGCTCGTCGATTAGCCA
ATGCTTTTTCAGGTAAGCCAAAAGAAGGGGAAATGTATTTTTTGGATTTATCGGAAGATC
AAGAGCGTTCTAAACGCTATAAAATTATTGAAGATTATCTTTCTGAAAAAGACTACCTCC
AACCCAAAGTCCCCGACTTAGACGAGATCGTGCCATTGCCACCGGCGCCCCTGCCCGATT
GGGATGGCAAAATCGCCTTTCAGCGCTGGTTTGAAGGAGAAGCCCCGCCGAAACCGAGTG
AAGCACTGATGTTCAAACTGGCAAATCAGGCAGGCGTTCGGGTGGATAACGGATTGGATT
TACAGACCGATTTGCCGAAGGCAGTGAAAAAATAACGAAAAAAACCACCGCACTTT

>REPEAT08
CCACACAATCCAACTACCGCTATACCCTGGATGCCGGTGAAAAGCACCATTTTGATGTAG
TGAGCTTCAAGCTGAGCGAAGGGCTGTCCGAACCCTTTAGGTTGGAGCTGATGTTATCAA
GCATTGACCCGAATGTGTCTTTCTCAGCCCTGATGGATAGCCGGTGACCTTCACTTTCCG
GCAGGGGGATAAAGCGGTACGCTATTTGAACGGGATTGTAACCGGATTAGGCGTGGGCAA
CAGCGGCTTTGTGCGCACCCATTATCACATGGTGGTGGAGCCGGCATTGGTGCGCAGCAT
GCTGCAATCGGACAGCCGTATCTTTCAACATCAAAACAGTGAAAAAATCATCCGCACGTT
ATTGCAAAAAAGCCGTGTGGATAAGGTGGCATTTGCCCCCTTGCCGGCGGATTGGGAGCG
GGAATATTGTGTGCAATACCGGGAAACGGATTTTGCTTTTATTGAACGCTTAGCGGCAGA
AGAGGGGTGGTATTATTATTTTGCGCATAACGCGGATAGCCATGAACTGCGTTTTGCGCA
TCAAAGCACGGCTTCACCGGTTTTAGGCACGCTTACTTATAACGGTAATCCCGCCGGCAA
CCGTCCTTTCGCCGGCCTTTGGCAATTTGAGTATTGCCGTCAAGTGACGACAAACAGGCA
AACCTTACGGGATTACACGTTTTTAAACCCGAACTATAATCTCGAACATCAGCACAGCTC
ACCATCCACAGCCCTCACCGATAACAACCGCTCAACTCATGCTCAAAGTGCGGTCAATTC
TCAAGATGTTTCACATTATGAAAAATACGATTACCCCGGCAGATATCAGCGCGACGAACA
GGGCAAGCAGTGCAGTCGTTATCGCTTAGAGGCAGAAATAGCTCTGTCGGAAACCGCCAA
TGCCACGGGAGATGATATGCGGCTAATACCGGGTTATGGTTTTACTTTAGAAGGCCATAT
CAATCCTGCATTTAACCAAGATTGGCTGGTAGTAGGTGTTGAACACACGGGCTGCCAAAC
AGGCGTGCTTGAAGAAGAAGCGGGTGAAAGCGGTAATTATTATGAGAACAGGCTCTTTCT
GATTCCGCATCACACCTTATGGCGCAGCCCGCAAAAGCCGCGTCCGGTTATCCGTGGCAC
TCAGGTGGCCCATGTAGCGGGCCCGCAGGGGGAAGAAATCTATTGTAATCAATGGGGCAG
CGTGAAACTGCAATTCCCGTGGGACAGAGAAGGCAGATTTGATGAGCACAGCTCCTGCTG
GGTACGTGTAGTACAAGATTGGGCAGGGGCACAATACGGCAATCTGATGATACCGCGTAT
TGGTGATGAAGTACTGGTGAAGTTTTTAAACGGCGACCCGGACAGGCCGATAGTCACAGG
CAGAGCCTACCACAGTACCACCGAACCTCCCTATCCCCTTCCCGAACATAAAACGCGCAT
GACGATTAAGAGCAAAAGCCACAAAGGCAACGGCTTTAATGAACTGCGCTTTGAAGATGA
GAAGGACCTGGAGGAAATCTTTCTTCATGCAGAAAAAGACTTAAACCATATCGTCAAACA
CAATGAAACCAGCCAAATCGGCCATAACCGTACAGAGCAAGTCAGCCGGGATGAAACCAT
TCACATCGGCAACAACAGAACGGAAACCGTAAGCCAAGATGAAGACCTCACCGTCAAGCG
CGACCAGACCCGCAACATCGGACGTAACCGCATTACCAAAGTTGAGCAAGATGAGGTTTT
AAATATCAACAACAACCGCTATGTCAATGTACATGGTGACACCATTATCCATGTGGGCAA
AGAATTGAATATTGAAATCGCACAAAACGGCAGTTGGCATGCCGGTGAGCTGTTTGAGCA
AATCTGTGAGCAGTTTGAGCTTGAGGGTTATGAGCAGGTGGAACTCAGTGGCCCGGGGGG
TTCGATAATTCTCAATCGGGAAGGGATAACCTTGGTGGGGGATGTGTATATTGAGGGGGA
GCTGGTGATGGAAGAGGAAGAGATGGAG

>REPEAT09
ATGTCAGAACAAAAAAAGCAAACAGTACTAAATCTAACAAAACATAACCAATTTGAATCC
CCCGAGATCCCTAACGTGGCTTATCCGCTAAAGCCTAAAAACAACACAAATGTATCCCAA
CAATATTTTAATTGCCTTGCCGGCGATGAAAGCGCAAGATTTCTGTTTAACAACAGCGGA
CTTTGGCACCAGGGGATTCATTTAAGAGCCTCGAAGTTTCCGGGTAGTGACTTTGAGAAC
GATAAGATTTGTGCTATTGCCGATGGCAAGCTGATTGCTTATAAAGTGGATAGTGAATAT
AAAAAGGACAGTGAAGTTGAAGTACCGATGAAAAGTGCGGTGTATTCTACAGGCTTTTTT
TTGTTGAAGCATGAAATGGCCTATCCGAAAGACAACGTACTCACTTTCTATTCCCTCTAC
CGCCATACAGCCAAATTGACGGATTATCCACCGCCGAAGCGTTATATCACCAAATCGGCA
GATGCAAGTCCGGTGGCACTCAAAGACAGACGGGGCGTGGTGATTGCGCAATTAGCCGAT
GGCCTGGTAATTTCCATCAAGTCCCGAGAGCGCAAAGCGTACCGTCACGAACTTGAATCT

TATCAAGATGAACAAGGCGTGATTCACCGTCCGCCGAATGGAGATATATGGACGATTTAT
AAAGGCTCCTATTATCAAGAGGAAGAGAAGGGTAAGCATGCTATTCCGGTTTTATCGCAA
CACAATATAGAGACGCAGGCGGATAAGGAAGTGCTCCTGAGTGGAGCACAACAAATCGTC
GTTAAGGCAGGTGAAGTGTTGGGCTTAATGGGCGAATATAACCAAATGCGGGAAAGCGGA
GAAAAGCTGTTTCAGCTTGAAGTATTTACCTATGACAACATGGAGCAATTTAAAAGCAGG
GCGGAAGCGGCTTATAAGCGGGATAAAGAAAAGAAAGGGCTAACAGATAATTTTCTGTAT
GTGGCACGGGGCAGTTGGCTTTATACGATACTTAATGGTGAAGCGGTGGAGCTGGAGAAA
ACGAAGGTTGAAATCATGGTGCCGTTATCTGATGTTACAAAGCAGACGGTGAAAGAGAAA
CAaAATCCGCAGGAAACGAAAGCATATtACAATGTACAGCCGTATCTTTAGAATCTGCTC
CAAAACAATTCAGACAATGGAaTTTATaTGGATGACAGCCATGTCAcgCATGGTATTTTA
TTTCCtGGGGTGAATGTATTTAAAGATGAAGCCCATGAAGTGAGTATTTTTAAGGAAAAA
ATCGCCGTACGCGCAGACCCTGAGAGTAGAGCAACGGCGGAAGAAAAAGACCGATTGGGT
GCGGTATTTAAGGAAATTTGGCAGGAGCTGTATGCGGGCAAGGATGGCAGTGgCGGAGAa
CaggggAAGCAAGATTTGAGGCGGGCAGTTTAAAACAATTATTGACAAACCCGCTAACA
GGCAGACGGCTGACGGGGATAGTCGTGAGACATAAGAGTGAGTGGTCGGTGGGACAGGCG
GGTAAGTTTGAAGAGCTGAAGGC

>REPEAT10
CCGCACTTTTATGCCTAATGTCAGAGCAGCGAAATCAAAATCGCTGCTCTTTAAATAACA
GATAACAAAAACCCATAACACCATGACAGAACCGCAACTCTGGGTGGGCTACTATCAGCT
CACCTATCTCACTCAACCGCAATTTACTTTGGTCATCTTATCGGCTTTGCCCGAAATCGT
GATGATTTTCAGCAAAAGATTGAACATTATAAAACCACGCATCAATGTAAACTGGTCAGC
CAGCTTGTGCCTTTACCGGCCCTCACGTGGTTTCAGCGACACGGACACCAGGCCCTCATT
TTTGCCCAAGCCCAACAATTAAAAGAAGAGGAACTGCGTTTTTATTTGCCCGGGAAGAA
AACCACCGTACCGACGAACAAAGCTATCTGATTGAACAAAGCACCACGCTCTCCCCTTTT
GTTCAGCCAACACCGCGTCATCAAGGTGTCACATCTTATCTGCCTGAAAAACTGTCAACC
CTTCCTTTCTTTGAACAACTCACCTCCCGACATAATCCTAAAGAGGGCTTGTATTATCGC
AATCCGGCATTTATTCCGGGTGAACAAGATGCACGTTATTATGCCATTATTGACGGGGTA
AAATCCTTTGTGTTGCCACAGTTAAGTGAACATGAGGGCAAAACCGATTCACTGTATAAA
GGTGAATTAAAAGAAAAGATGGATACCCATGCGCCTTTTCTTGCCCAACTCACCGTGACC
GATAATGAAACCAGCCCCTTTGTGCAGTTATTGTTTAGTCAAGCCGAACAACCCTGGTTC
GGTGCTTGGGATATTAATCCTGCGATATTTATCCGCACCACGCAAAAGTTTGACACCCTC
GCTTATCACTTACGTAAATTTATTCATTTATACAATGAGCAAACGGGAAAATGGTATTTT
TTCCGCTTCTATGACCCACTGATTCTTGTGGCATACCTGCGTTATATCGCGCACTCACCG
GATAAACTCGTGTCTTTTTTTGGCATAAGAGACGGACAAGCGATGATAGCATCGCTCGCC
GCCCGTATTGGCAACCAGTTTTATACTTTTTGTCTTGATGCCTTACCGGAAAACACACAG
CCGGGCACGGTGAGTTTTGATGAAGAATTTGAGGCGTTTTTGATGGATTATGACAAACAT
CAATTGCTTGAAAAGTTACAGAAGGAGATTATTCCGCAAGAATTTGGCAATAATAAAATA
TCACCGCCGGAGATTGAGAAATATTTTGAAAAAACACTACAAGCGGGCTTTAAA

>REPEAT11
AAGTCTGTAGTGATCAACTAATTCAACAGTCCCCGATAGGTTGTTTTTGTTAAAATGGGT
GGCAAGCCTTGATTATAACGATGTGGGCGAACAAAATTGTAATACATTACATAATCCCTC
ACATCGTCTATCGCCAGCCCGAAAGTTAAATAGCCGCCCTCTTGCATCCACTCATATTTA
AAACTGCAGAACCAGCGCTCCATTGGCGCATTGTCCCAACAATTACCACGGCGACTCATG
CTTTGGGTTAAACCGTAACCTTGCACGGTTTTGCAAAATACCTTGCTACCATAAATACTG
CCTTGTCTGAATGGAAAATCATTTTCGCCGTCTTTTTGACGTTCAGCATTGCGTGATGCA
ATGCCTCGACAACTAATTCACTGTCGTGATAACGACTCAGCTTCCAGCCTACCACTTGTC
GGTTAAAAAGATTAATCACCACCGTTAAATAGCACCAAATCCCATTTACTTTAAGATACG
TCGTATCGCCACAAAGCACCGTCGTATCCGCATTCGGTGTAAATTCTCGGTTCAAATGAT
TCGTAAAAATCTGCCCATTCTCTTTATTTCGATTTTGCCATTTTTGCGGTTGTTTGCTGA
ATAATCCCTGCTGTTTCATCAATTTTTGAATCAAATACAGCCCAACAAAGATAcTTTtCT
GTCTCAATAAATCACGTAATGTTCGCTTGCCTGCCGAGCCACGACTTTCAACAAATAGAG
ATTTAATTTCCGGTGAAAGTGCGGTGTGTTTTTGTGGCGTTTTTACCGCTTTACATTGGG
CATAATACGCACTTTCAGACACCTCAAACAGCGCACAAAGTCGTTTGATTCCGCGCGATT
TTAACGTCGTGATGGCTTGGTACTTTGCTTCTCGAGGGATAAATCGCGGTAGCTTTTTTT
AGAATGAGCTTGTCTTCTTCCAATTCTTTGATGCGTTTCTCCAACTCGTAAATACGTTAT
TGCTCGGGTGAAATCGGTTTGCTGCCGGGTAAGACATAACCTTGATTCTCCGCTTCAACC
TGGTTTATCCGGCGACGCAATACCGTTTCGCCGATGTCTCATTCTCGGCAGGCTTGTGCA
ACCGAATAACCACGTTCTTTGATTAATTTGACCGCTTCAGCTTTGAACTCCGGGCTGAAA
AAACGTCTTGCTCTTTTCATTATAAAATCCTCTTGTTGATgaTATTTTACCACTTATTGA
GGAATGCGGGATTAGTGTACCACTACA

>REPEAT12
CCAAGTGCAACGATTTTTTAAAGTAAACTTCATAAGGTGTTTTCCAGCCTAAACACTTTC
GTGGTCTTAGATTGAGTTTATCTACGACAGACTGAATATAATCATCGTTCCATTGGTTTA
TATCCTGTTGGTTCGGAAAATATTCCCGAAGCAACCCGTTTGTGTTTTCATTCGTTCCCC
TTTGCCACGGCTGGTGCGGCTCGGGAAAATAAAATTCCGCTCCCAATGCTTGGGTTACCA
AACGATGTTTGGCAAACTCTTTGCCCCTGTCCAGCGTAATTGACCGCACATTGTGGAATT
TTAGTAATGTTATCGTCGCCTCTTTGACCAACATAGCTTTTTTCGCCGACACTTTCATGG
CTAACTCAAAGCGACTTTTCCGTTCGGTGAGTGTCACTAAGCAGGCGCCTCCCACCACAC
CTAACACCGTATCCGCCTCCCAATGTCCGAATCGACTCCGGTTCTGCGCCGAAATCGGAC

GCTCGTCCAAGGTGTGGGAAATCACGATTTTTCCGCGTGTTTCCAAGTGACGTTTGGTGT
GTCTTGTCTTGTCTTGTCTTGCCTTTGTGGCGAAGTTTTCGGGCAGCTTTTCGTTCACCA
ATATCAAACCAACCTTGATGAACCGCACGATAAATCGTGGAATAACTGATTGATAAAGGC
GATTTCTCTAATTTCAATCGTTGACTAAGCTGTTCGGGAGACCATTTATCCAGCAGTATT
CTGTCTTGCACCAAGGTGCGGTATTCTGCACGTTGCAGCTTAGGTATACTTTTGCATTTT
TTTCGATGGATTTGATAACTCTGTTGTGCTTGACTTGCGCTATATACCTTAAGTGAATGC
CGTTTTAACTCACGGGATATGGTACTGCTGCTTTGCCCTAATCGTTGTGCAATTTCAAGT
TGTTTTTTACCTTGAGTGCGCATAATCATTATGCTTTCGCGCTCTAAAAGAGTAAGATGT
CGATAAGAAGAGTTCATTTGCGTGTTTGTTTTTTGTTTGGGAACAAAAATTATACACAAT
TATGAACTCTTCTTTTTTGCACTTGGATTGTAAATTCAAG

>REPEAT13
TCGATACGCCTTTTCAGTTTTACTTTGGTTTGTCTTATCAATTATTTGATAATTTTAGCT
ACCACGCCGGCACCCACTGTACGGCCACCTTCACGGATAGCGAAACGTAAACCTTGGTCC
ATCGCAATTGGGTGAATTAAGGATACGGTCATTTTGATGTTATCACCGGGCATAACCATT
TCCACGCCTTCAGGTAACTCGATAGTACCGGTTACGTCAGTTGTACGGAAATAGAATTGT
GGACGGTAACCTTTGAAGAATGGAGTATGACGACCACCTTCTTCTTTGGACAATACGTAC
ACTTCAGATTCGAAGTCAGTGTGCGGGGTGATTGAACCCGGTTTCGCCAATACCTGACCA
CGTTCGATTTCTTCACGTTTAGTACCACGCAATAATGCACCGATGTTTTCACCCGCACGA
CCTTCGTCAAGTAATTTACGGAACATTTCAACACCGGTTACGGTGGTTTTTGCAGTCGGT
TTGATACCCACGATTTCAACTTCATCACCGGTACGGATGATACCGCGCTCAACACGACCC
GTTACTACGGTACCACGACCGGAGATAGAGAACACATCTTCAATTGGAAGAAGGAACGGT
TGGTCGATAGCACGCTCAGGTTCCGGGATGTAAGTATCTAAATGGTTTGCTAATTCAAGG
ATTTTTTCTTCCCATGCGGCATCGCCTTCAAGCGCTTTTAATGCAGAACCGCGTACGATT
GGGGTGTCATCGCCCGGGAAGTCATATTGAGAAAGAAGTTCACGAACTTCCATTTCAACT
AATTCTAATAACTCTTCGTCATCTACCATGTCGCATTTGTTTAAGAATACGATGATGTAA
GGAACACCTACTTGGCGACCTAATAAGATGTGCTCACGAGTTTGTGGCATAGGACCGTCG
GTTGCTGCTACTACTAAGATAGCACCGTCCATTTGCGCCGCACCGGTAATCATGTTTTTC
ACATAGTCGGCGTGTCCCGGGCAGTCAACGTGTGCATAGTGACGGGTTGGGGTGTCATAT
TCAACGTGTGAAGTGTTGATGGTGATACCACGCGCTTTTTCTTCAGGTGCATTATCGATT
TGGTCAAATGCACGTGCTGCACCGCCGTAGTGTTTTGCCAATACGGTAGTGATAGCTGCT
GTTAAAGTGGTTTTACCATGGTCAACGTGGCCGATTGTACCCACGTTAACGTGCGGTTTT
GTACGTTCAAATTTTTCTTTAGACA

>REPEAT14
AAAAAAACCACTTACCCCTCTCATCCTGCAACACCGGAAATTTACGCAGATGGTGGTAAA
CCCTGTCAAAATCAGCATATGAATGGATAAAAATACCTAGATTATCTTccAGTTAAAATC
ATGAAGTGCTGATTTTAAACTGAATAAGCCCATCATTTCACCGGTGTGAATGTGCTCTTC
ATCGCGAATAACTTCGATTAAATAAGGCGCCACCTTTGCCGTGTCGTCGCCAAACCTGCC
TTGAAACAGGCTTTTCATTTTCGCTTTCAGGCTTAATGTGTGGAAGAACGGCATCTTATT
GGCATCTAATACAAAATAGGTTTTTAGCTTGGGTAACGGATAGTTCTCCCCGCCCTTTCC
CTCTTCTCCGGCATTTTCAGCATTTTTACCATATTGTGACAGATGACGCGCTTTCACCCT
TTCATCAATGAGTGGTCTTGCAAAACTCAACTGCACCGGCTTGTCTTTATTGGCAAAATC
CAACAAATTAGGCGCAATAGCAAAATCCGGCCATAGAAGCTGTGCAATGGCTTCAGGAGC
AAAAAGTGCCGGTATGCGGTCAAGCTGCATATCAAGCGGTTTGATATTGTCAATAACATG
GCAATGCAAATACGTAATATCGCCTTCTCTTACCGGTTGCGGCGTTTCATCATTTAAAGC
AATGACGCGCAGTTCATCTTCTGCAAGTGAACCAGCTTGATGAAGCAACCAGGTTCTTCC
ATGTCGTTGAAAAAaGACTTCGGCTTTTAGGGGCGCCAGTTGGTAATAATAAGGTATGTT
CTGCGCATGGAAAGTGGATTTTACTAACTGTTCAAAAGCATCTCTGCTCTCGGCAAA

>REPEAT15
CCAGCTCCCCCCGTGAACGAGGGGAGCCAAgtttttttttCTTTTTATATAagTAGTtAGG
GCGTGTTGATCTTTCCAGTATAAAAAATAAACGACATAGAAGTTTGGTATAATTGAGTCC
GCAAAAACCTGAATAAACCTTACAAACTATGTCGTTGAGAACTATTTTAACAGATAAGCT
GTGGGATCGCCTGGcTTATTTACTTGACCGAACAGGTCGTGTTTACAATAAACCCGAACA
TCGCAaTACCGTCGAAGGTATTCTGTACCGTCTTCGCACAGGATGTCCTTGGCGTGATTT
ACCTGCAGAATTTGGTTTGTGGAACACCGTTTATCGCCGTTTCAATCTATGGTCAAAGAA
AGGTATTTGGCAAGAAGTGCTAAAGTTCTTTCGGCAGTGTATTGATTCTGAATGGATTTT
TATTGATGGCAGCATCGTCAAAGCACATCAACACGCAATGGGGGCTAGCGGTCAAAATCC
GCAAGCTATTGGCAAAAGCGTAGCGGGCAATACGACGAAAATCCATTTGGCAGTGGATAG
TTGTGGTAATCctATCGACTTTATTTTAACGGGAGGCGAAGTTCACGACAGCAAaGCCGC
ACCTGATTTAGTGGCATTATTGCCTGATAGTGAAGCGATTATTGCAGACCGCGGATACGA
CTGCCAAGCCTTGCGAGAGCTGATTTTAAC

>REPEAT16
AGTTTAAAGACGGATAAACTTTATTGTGAAAACGATGCCATTTTGTCTTTGTGATATGAC
AGAATTTTGATTCACTATAACTTTTCAAATTTTCGGGTGGACGATAAGCTCCCATACCAA
CGGCGGAAAACGAAAATACCGGTTTTCTGTGTAAAGATAGCTGATGGCTGTTTAATGTTT
TTTAAAAGCATGAAAAACACCATAAAAATCCACCGCACTTTAGACTAAAGTGCGGTGGGG
TTTTTCGTTATTTTTTCACTGCCTTCGGCaaatcGGTCTGTAAATCCAGTCCGTTATCCA
CCCGAACGCCTGCCTGATTCGCCAGTTTGAACATCAGTGCTTCACTCGGTTtcGGCGGGG
CttctccTTCAAACCAGCGCTgAaaGGCGATTTTGCCATCCCAATcgggcaatggagcag

gTGGCAACGGCACGATCTCGTCCAGATCCGGGACTTTGGGTTTGAGGTAGTC

>REPEAT17
GCGTTTGGCGATTCAGCGCAGAGCAAGCGAAACGGGGCTTGCTGTTTCATTCGGACAGAG
GGGCCGGTATAGTAGTCGGGCATTTAGATAATATCCGGCGGATGTTGGTATAAAGCAAAG
TCAAGGCCGGGCGGGGAAATGTACCGATAATGCGGTAACagAGCGGTTTTTCCGCAGTTT
AAAGACGGAGAAACTTTATCGGGAAAACTATGCagCTAAAACTGCCGcACTGCTTGGTAT
TGCGGAGTATATTGAGgaTTTTTATAATCCGAAACGGCTTCATTCGGCATTGGGTAACTT
ATCACCGATGATATTTGAAGCGAAACAACCGTGTTTAACACTTTAAATATATCTTTGCTA
TGTTGCGGAATTTTGAACCATTACATT

>REPEAT18
ACAGCTGTTCGAAACATTATAATTCATTGATATTTTTAAATATCTTGCCTTCCTCCGTTT
ACGGGGgACAAAACGCATTGCGTTTTGAGCGTTGGCTCTGCCAACGACCCCAAAGGGGTG
AGCAAGTAaGTTTaGAaCTTGCGAATAACGTGCCCGAAGGGCGGAAGGGGGGATTTATCt
cGATGTTTTCACAAGATCATGCTTAAAAATTGAGATTTT

>REPEAT19
GACATCATTTTGCTGACTTGTGcGATGGTGTGATGTTGTTTAAGCATTAAATCGACACAT
TGTTGTTTGAATGTctgggTAAACAGTTTGGTCATgaGCTTTTCCTtCTGCAAATTTTAA
TTGGTTTATCAGACTGTCTTTGCGGTGTTGCTGAAAGTTAGA

>REPEAT20
AAAAaaTATTCATAAATCACTGAAGTGATTTATTCACCCCTtCGGGGCCGTTGGCatAGC
CAACGTTCAAAAAACGGaaTTTTTTGTGACCGCACTTTTG

>REPEAT21
GCACGTTCGCGTTCAATACGGGCATTTTCTTCCTGCTGACGTTgtGcttcaatcgcGCGT
TGACGTTCTTCTTCCACTGCTTTTTGACGGGCgaTTTCCTGTTCACGGGCTAGATGGTCG
GCTTCTTCACGTTTT
